# Coumarin bearing triazole hybrids as cholinesterase inhibitors targeting Alzheimer's disease

**DOI:** 10.1039/d5ra09311b

**Published:** 2026-04-20

**Authors:** Shahab Kermaninia, Morteza Farnia, Mohammad Mahdavi, Aida Iraji

**Affiliations:** a School of Chemistry, College of Science, University of Tehran Tehran Iran mfarnia@ut.ac.ir; b Endocrinology and Metabolism Research Center, Endocrinology and Metabolism Clinical Sciences Institute, Tehran University of Medical Sciences Tehran Iran; c Stem Cells Technology Research Center, Shiraz University of Medical Sciences Shiraz Iran iraji@sums.ac.ir +98 713 230 2225 +98 713 230 3872; d Research Center for Traditional Medicine and History of Medicine, Department of Persian Medicine, School of Medicine, Shiraz University of Medical Sciences Shiraz Iran

## Abstract

A series of novel coumarin-triazole hybrids (12a–s) were synthesized and evaluated for their inhibitory activities against cholinesterase including acetylcholinesterase (AChE) and butyrylcholinesterase (BChE), enzymes related to Alzheimer's disease. The structure–activity relationship (SAR) analysis revealed that substitutions at the 2-position of the aromatic ring significantly enhanced anti-BChE potency, with compound 12c (2-fluorophenyl) exhibiting moderate activity with IC_50_ = 4.37 ± 0.91 µM for BChE and 7.17 ± 0.42 µM for AChE. Docking studies demonstrated strong binding interactions of 12c with critical residues in the active site of the enzyme. Molecular dynamics simulations confirmed the stability of the 12c-AChE and 12c-BChE complexes over 100 ns, with low RMSD values and stable hydrogen bonding. These findings highlight the importance of electronic and steric effects in optimizing cholinesterase inhibition and provide insights into the design of effective agents for Alzheimer's disease therapy.

## Abstract 2

A series of novel coumarin-triazole hybrids were synthesized and evaluated for their potential as anti-Alzheimer's agents.

## Graphical abstract

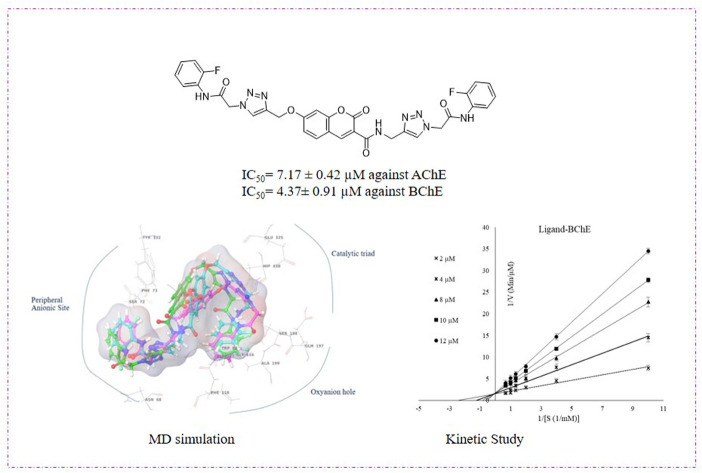

## 1. Introduction

Alzheimer's disease (AD) is the leading cause of dementia, responsible for 60–80% of all cases. Given that age is the most well-known risk factor, AD poses a serious public health concern for the elderly. After the age of 65, the frequency of AD doubles every five years, and by the age of 85, almost one-third of people may have this disease.^1^

The global prevalence of AD is expected to rise dramatically with increasing life expectancy, placing an enormous burden on healthcare systems and societies. The primary symptoms of AD are memory loss, cognitive decline, and behavioral problems. AD progresses gradually, causing symptoms to worsen over time and ultimately making routine tasks more difficult.^2,3^

The disease is characterized by an accumulation of amyloid-beta plaques and neurofibrillary tangles in the brain, which lead to neuronal death. This neurodegeneration leads to brain atrophy, affecting memory and learning-related regions including the hippocampus and cerebral cortex.^4,5^ Also, according to cholinergic dysfunction, the decline in acetylcholine, a neurotransmitter crucial for cognitive function, is a major feature of AD pathogenesis, which exacerbates cognitive function.^6^

Two key enzymes involved in acetylcholine metabolism are acetylcholinesterase (AChE) and butyrylcholinesterase (BChE). AChE hydrolyzes acetylcholine at the synaptic cleft, stopping cholinergic signaling. In Alzheimer's, there is a reduction in the level of acetylcholine due to cholinergic neuron loss, which plays a major role in memory impairment and cognitive decline. Inhibition of AChE can help increase the acetylcholine level, hence, supporting cognitive function.^7^ Although acetylcholine is mainly broken by AChE, some studies indicate that BChE activity is increased in the brain of AD patients, particularly at the late stage of the disease. It was proposed that BChE contributes substantially to the remaining cholinergic activity and acetylcholine breakdown in advanced AD. As a result, pharmaceuticals that target both AChE and BChE have emerged to be effective strategies for enhancing cholinergic function and lowering cognitive deficits.^8^

Currently, there is no cure for AD, however, current medications try to reduce symptoms.^9^ Despite extensive efforts, available drugs such as donepezil, rivastigmine, and galantamine offer only modest symptomatic relief without altering disease progression. As life expectancy increases globally, there is an urgent need for further research to develop more effective therapies for the elderly. Scientists have put enormous efforts in developing effective drugs to target this disease through developing cholinergic inhibitors.^10–12^

**Fig. 1:**
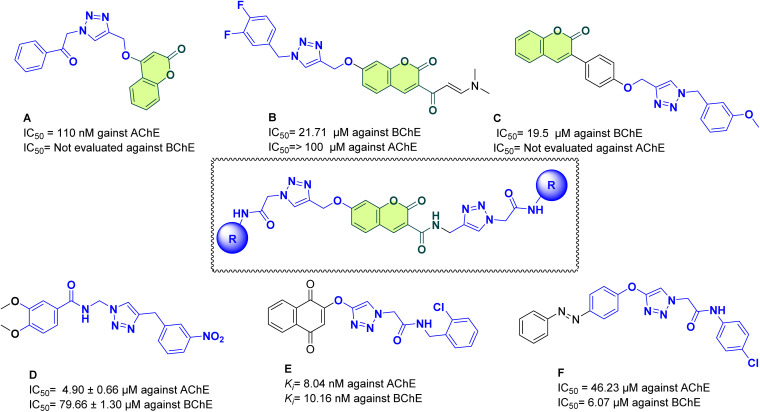
Designing strategy.

Coumarin-based compounds have attracted considerable attention as potential small-molecule candidates for AD therapy due to their diverse biological activities. Coumarin scaffold as synthetic and natural compounds is widely present in different structures as may possess neuroprotective, anti-oxidative, and AChE inhibitory potency, it is reported that owing to the presence of polar elements in the structure might facilitate better interaction with the biological target.^13,14^ Bhagat *et al.*, in 2021, used a rational drug design approach, and a new series of coumarin or isatin scaffolds was developed. The AChE inhibitory activity showed that coumarin-1,2,3-triazole hybrids (compound A, Fig. 1) were noted to be more powerful inhibitors than isatin-1,2,3-triazole hybrids. Structure–activity relationship (SAR) studies revealed that also presence of an electronegative group such as F, Cl, or NO_2_ on the substituted region improves AChE inhibition.^15^ Likewise, 1,2,3-triazole-chromenone compounds (compound B) shown encouraging anti-AD effects, with an IC_50_ of 21.71 µM against BChE and significant suppression of self-induced Aβ1–42 aggregation and AChE-induced Aβ aggregation (32.6% and 29.4%, respectively). According to SAR analysis, activity was increased by small-size halogen substituents.^16^ The 1,2,3-triazole ring is a versatile scaffold with advantageous properties such as stability in both basic and acidic environments, excellent bioavailability, and low multidrug resistance.^17^ Effective inhibition of AChE and BChE activities is made possible by its properties, which promote enzyme–inhibitor interactions. Docking studies have shown that triazole-based derivatives, including dimethylaminoacryloyl-chromenones (compound C), and aralkylamide (compound D) have strong anti-ChE activity, with van der Waals and hydrogen bonding interactions in the enzyme's active region being highlighted.^18–20^

Recent advancements include hybrid of triazoles such as 1,2,3-triazoles-naphthoquinone, where *ortho*-chlorine derivatives exhibited strong inhibitory activity. This derivative forms multiple interactions with the catalytic triad and the peripheral aromatic site (PAS) of the enzyme. Among the synthesized derivatives, compound E, featuring an *ortho*-chlorine moiety, exhibited the strongest inhibition of AChE and BChE, with *K*_i_ values of 10.16 nM and 8.04 nM, respectively.^21^ More recently, phenyl-diazenyl-phenoxy-1,2,3-triazole-acetamide (compound F) has emerged as a novel scaffold developed through the molecular hybridization of active pharmacophores. *In vitro* evaluations revealed that all newly synthesized compounds were more potent inhibitors of AChE and BChE than the standard drug galantamine. According to molecular docking experiments, the strongest compounds formed interactions with important elements of the AChE and BChE active sites, such as PAS and the catalytic active site (CAS).^22^

In this regard, the goal of the current work was to use molecular hybridization to develop new active molecules. A novel set of compounds with coumarin-triazole acetamide scaffold were designed, and synthesized, and their *in vitro* and *in silico* efficacy as possible anti-AD agents were assessed. A kinetic study was also conducted to learn more about the inhibitory mechanisms of the strongest analogs. *In silico* evaluations, such as molecular docking and molecular dynamics simulations, were carried out to predict and validate the binding interactions of these pharmaceuticals with their target enzymes to support the experimental results.

## 2. Results and discussion

### 2.1 Chemistry

**Fig. 2:**
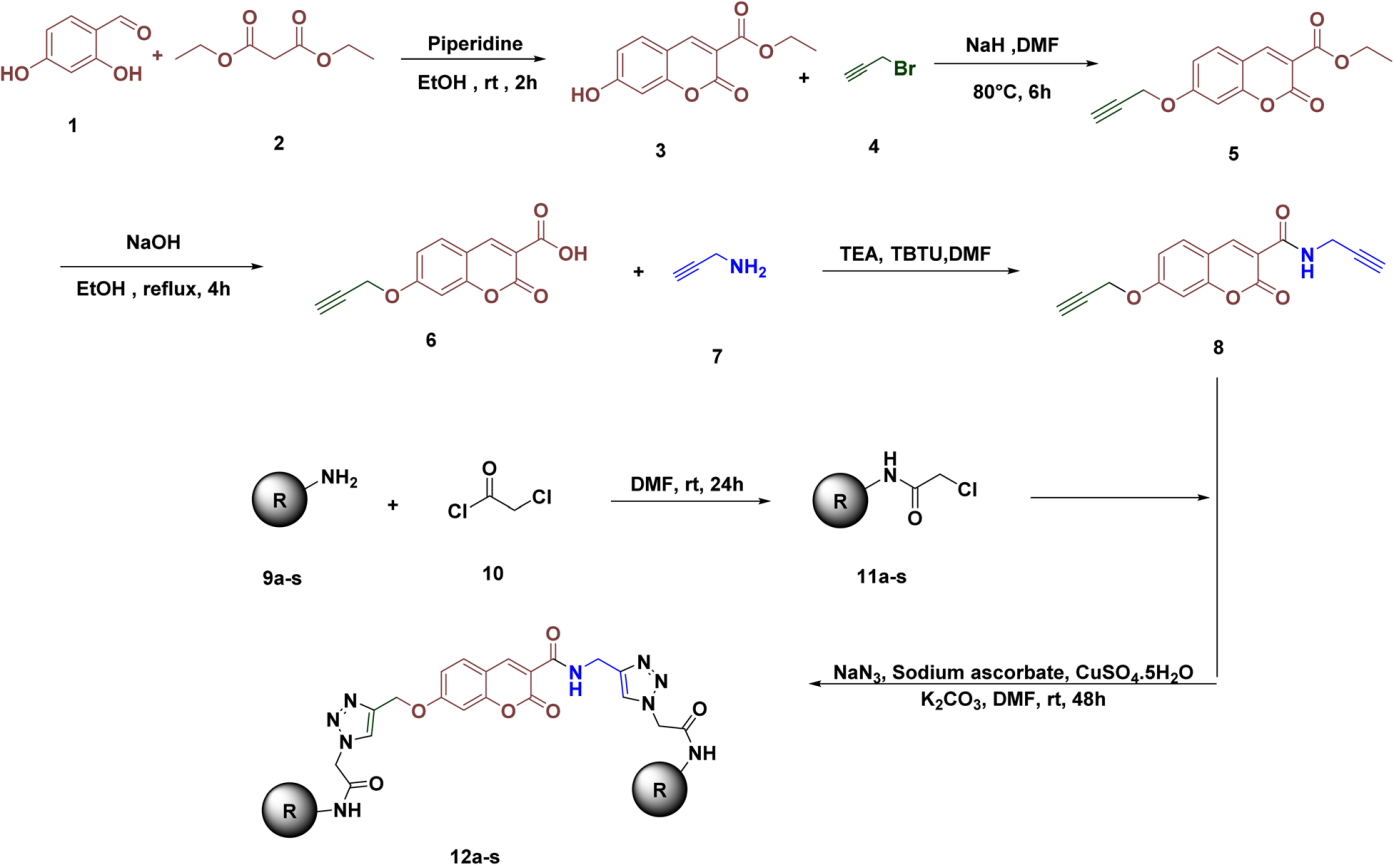
Synthesis of 12a–s.

A synthesis of triazole hybrids 12a–s is exhibited in Fig. 2. Briefly, 2,4-dihydroxybenzaldehyde (compound 1) was reacted *via* condensation with diethyl malonate (compound 2) in the presence of piperidine to form a key compound 3. Ethyl 7-hydroxy-2-oxo-2*H*-chromene-3-carboxylate (compound 3) underwent propargylation using sodium hydride and propargyl bromide (compound 4) in DMF at 80 °C, followed by precipitation and recrystallization from ethanol to produce the propargylated chromene derivative (compound 5). The product was then hydrolyzed under reflux in a 1 : 1 ethanol : NaOH (5 M) solution at 100 °C, acidified and extracted using ethyl acetate to furnish the corresponding carboxylic acid derivative in excellent yield (compound 6). Subsequently, the acid derivative (6) reacted with amine (7) in the presence of pyridine and in DMF to produce the carboxamide intermediate 8. This intermediate is then reacted with 2-chloro-*N*-phenylacetamide (11a–n) in DMF, sodium azide, copper sulfate, and sodium ascorbate, using click chemistry at 40 °C for 48 h. The final triazole hybrids are obtained by filtering the reaction mixture and washing the solid with cold water/ethyl acetate, yielding pure triazole derivatives 12a–n. The yields of the synthesized final products are presented in Table 1.

**Table 1:** Yields of the synthesized derivatives

Compounds	Yield%	Compounds	Yield%	Compounds	Yield%
12a	67	12h	68	12n	74
12b	78	12i	77	12o	75
12c	65	12j	74	12p	73
12d	61	12k	68	12q	66
12e	71	12l	72	12r	73
12f	63	12m	75	12s	71
12g	79				

**Fig. 3:**
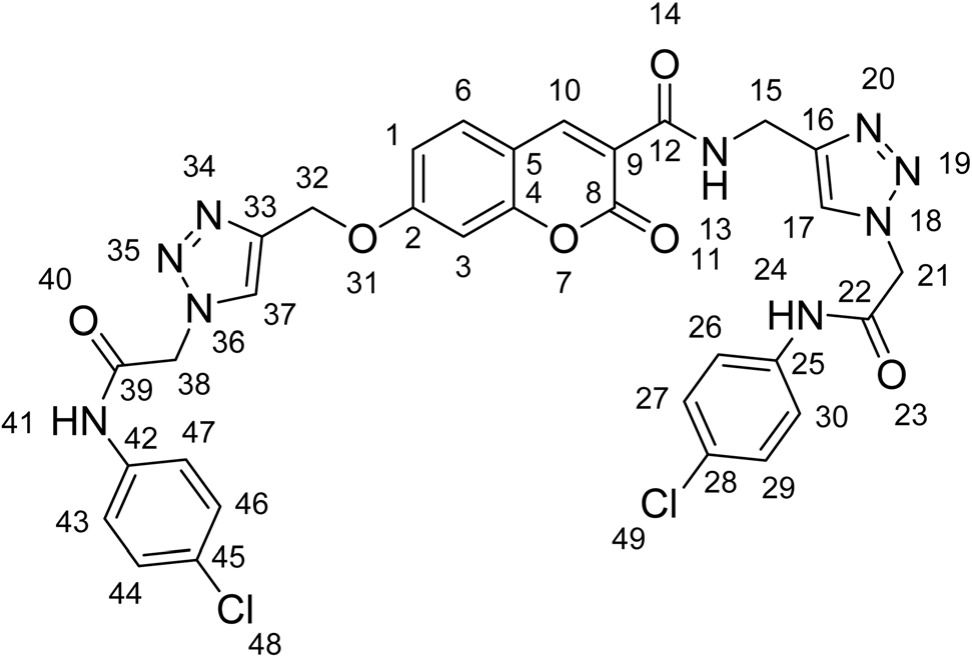
Chemical structure of 12k.

The structure of compound 12k was confirmed by ^1^H and ^13^C NMR spectroscopy (Fig. 3). Two signals at 10.62 and 10.59 ppm were assigned to the amide N–H protons (H24, H41). The peak at 9.10 ppm, corresponding to the other amide N–H proton (H13), appeared as a triplet due to coupling with the adjacent CH_2_ group. Two singlets at 8.33 and 8.88 ppm were attributed to the triazole C–H protons, confirming the successful formation of the 1,2,3-triazole rings. A singlet at 8.04 ppm was assigned to H10, which is located in the β-position relative to two carbonyl groups. The doublets at 7.94 and 7.31 ppm corresponded to H6 and H3, respectively, with H3 exhibiting a long-range coupling to H1. A doublet of doublets at 7.13 ppm was assigned to H1, which is coupled to both H3 and H6. Three singlet peaks at 5.31, 5.35, and 53.7 ppm were assigned to the three aliphatic CH_2_ groups. The doublet at 4.61 ppm corresponded to the CH_2_ adjacent to the amide N–H. In the ^13^C NMR spectrum, the most downfield signals were attributed to the two carbonyl moieties located at the termini of the molecular structure (C22, C39). The following four signals were assigned to four quaternary carbons directly attached to oxygen atoms. Two sets of high-intensity peaks appearing at *δ* 121 and 129 ppm corresponded to the aromatic carbon atoms C26, C27, C29, C30, C43, C44, C46, and C47. The most upfield signal in the aromatic region was assigned to carbon C3, due to the negative charge generated in resonance structures involving the adjacent oxygen atoms. The most upfield signal overall was assigned to carbon C15, which is directly bonded to the amide nitrogen. The most deshielded signal in the aliphatic region was assigned to carbon C32, which is directly bonded to an oxygen atom. Finally, two closely spaced signals observed at approximately 52 ppm were assigned to carbons C21 and C38.

### 2.2 AChE inhibitory activity

The relationship between structural modifications and biological activity against AChE was studied (Table 2).

**Table 2:** BChE and AChE inhibitory activities of 12a–n^a^

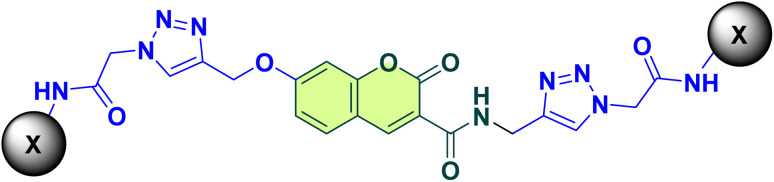					
Compound	R	AChE		BChE	
		% Inhibition at 50 µM	IC_50_ (µM)	% Inhibition at 50 µM	IC_50_ (µM)
12a	Phenyl	84.43 ± 6.56	23.06 ± 3.74	46.02 ± 0.41	>50
12b	2-Methylphenyl	52.59 ± 2.14	38.46 ± 0.63	56.19 ± 2.32	10.07 ± 1.63
12c	2-Fluorophenyl	62.03 ± 2.36	7.17 ± 0.42	66.66 ± 0.05	4.37 ± 0.91
12d	2-Chlorophenyl	33.92 ± 4.19	>50	54.71 ± 3.15	39.90 ± 3.89
12e	3-Methylphenyl	24.58 ± 1.47	>50	24.15 ± 2.63	>50
12f	3-Chlorophenyl	37.35 ± 3.52	>50	45.65 ± 0.55	>50
12g	4-Methylphenyl	15.73 ± 3.39	>50	37.98 ± 1.63	>50
12h	4-Ethylphenyl	16.44 ± 1.94	>50	39.29 ± 1.13	>50
12i	4-Methoxyphenyl	32.23 ± 1.48	>50	24.58 ± 3.36	>50
12j	4-Fluorophenyl	37.11 ± 3.02	>50	32.19 ± 2.93	>50
12k	4-Chlorophenyl	44.03 ± 6.83	>50	43.89 ± 7.41	>50
12l	4-Bromophenyl	21.75 ± 5.11	>50	39.38 ± 7.12	>50
12m	4-Trifluoromethylphenyl	19.33 ± 1.89	>50	38.78 ± 9.52	>50
12n	2,4-Dimethylphenyl	59.65 ± 1.77	6.65 ± 1.31	54.33 ± 2.46	17.07 ± 4.61
12o	2,4-Dimethoxyphenyl	52.53 ± 0.64	18.66 ± 1.82	58.37 ± 5.42	17.28 ± 0.70
12p	3,4,5-Trimethoxyphenyl	22.87 ± 2.36	>50	35.14 ± 2.46	>50
12q	Benzyl	56.24 ± 6.73	34.57 ± 15.74	47.50 ± 1.03	>50
12r	4-Methylbenzyl	54.53 ± 2.24	46.68 ± 0.89	10.49 ± 0.55	>50
12s	Phenylethyl	24.70 ± 3.24	>50	30.26 ± 2.41	>50

a Donepezil as positive control showed IC_50_ = 10.6 ± 2.1 µM against BChE and IC_50_ = 0.079 ± 0.05 µM against AChE.

The unsubstituted analog (compound 12a) exhibited moderate activity, with an IC_50_ of 23.06 ± 3.74 µM. Substituents at various positions on the aromatic ring had significant effects on the potency against AChE. Substituents at the 2-position showed mixed effects depending on their electronic properties. For example, compound 12c (2-fluorophenyl) demonstrated the most potent activity, with an IC_50_ of 7.17 ± 0.42 µM, highlighting the benefit of a small electron-withdrawing fluorine group. Conversely, bulkier groups such as chlorine (compound 12d) reduced activity, suggesting that steric hindrance may weaken enzyme interaction.

Substituents at the 3-position also resulted in weak activity. For instance, compounds 12e (3-methylphenyl) and 12f (3-chlorophenyl) exhibit significantly lower potency than the unsubstituted compound.

Most compounds exhibited moderate to weak activity for derivatives with substituents at the 4-position. Compound 12k (4-chlorophenyl) showed % inhibition of 44.03 ± 6.83, while compound 12g (4-methylphenyl), 12h (4-ethylphenyl), 12l (R: 4-bromophenyl) and 12m (R: 4-trifluoromethylphenyl) displayed even lower activity, with % inhibition values of 15.73 ± 3.39, 16.44 ± 1.94, 21.75 ± 5.11, and 19.33 ± 1.89, respectively. Also, 12i and 12j exhibited around 30% inhibition at 50 µM. These results suggest that the 4-position is less favorable for enhancing potency, regardless of substituent electronic or steric properties.

Disubstituted compounds demonstrated significant activity, influenced by the combination of substituents. Compound 12n (2,4-dimethylphenyl) exhibited the strongest activity, with an IC_50_ of 6.65 ± 1.31 µM, indicating that dual methyl substitution enhances binding affinity. Similarly, compound 12o (2,4-dimethoxyphenyl) showed an IC_50_ of 18.66 ± 1.82 µM, highlighting the favorable effects of electron-donating groups. These results indicate that 2,4-disubstitution is a favorable strategy for AChE inhibition.

The trisubstituted derivative compound 12p (3,4,5-trimethoxyphenyl) exhibited weak activity, with % inhibition of 22.87 ± 2.36, likely due to excessive steric hindrance and the reduced ability to interact effectively with the enzyme active site. Replacing the phenyl group with a benzyl group (compound 12q) showed moderate activity (IC_50_ = 34.57 ± 15.74 µM), but no significant improvement was observed compared with 12a. However, 12r bearing 4-methylbenzyl showed IC_50_ = 46.68 ± 0.89 µM, which showed improvement *vs.*12g.

In summary, the most potent inhibitors were compound 12c (2-fluorophenyl) and compound 12n (2,4-dimethylphenyl), which demonstrated strong activity due to favorable steric and electronic effects. Substituents at the 2-position generally improved potency, while those at the 3- and 4-positions showed limited or no enhancement. These findings underscore the importance of substituent type and position for optimizing AChE inhibition in AD studies.

### 2.3 BChE inhibitory activity

The relationship between structural modifications and biological activity against BChE was analyzed based on IC_50_ values and % inhibition at 50 µM. The unsubstituted analog (compound 12a) exhibited 46.02% inhibition at 50 µM, indicating moderate activity. To enhance potency, various substituents were introduced at different positions on the phenyl ring, which significantly influenced biological activity (Table 2).

Substituents at the 2-position generally improved potency. For example, compound 12b (R = 2-methylphenyl), with an electron-donating group, exhibited an IC_50_ of 10.07 ± 1.63 µM, indicating significant improvement over the unsubstituted compound. The most potent compound was compound 12c (R = 2-fluorophenyl), with an IC_50_ of 4.37 ± 0.91 µM, highlighting the effectiveness of a small electron-withdrawing fluorine group at this position. However, replacing the small fluorine group with bulkier groups like chlorine (compound 12d) reduced the potency. This suggests that steric hindrance diminishes the benefits of larger substituents.

Changing the chlorine position from the 2- to the 3-position significantly reduced potency to 45.65% inhibition at 50 µM *vs.* compound 12d exhibited an IC_50_ of 39.90 ± 3.89 µM for 12f. Similarly, 3-methylphenyl substitution (compound 12e) decreases potency relative to 12b. This observation highlights the destructive role of *meta* substituent position for activity.

Substituents at the 4-position exhibited moderate to low activity across all derivatives (compounds 12g–m), with no significant differences among them. This suggests that substituents at this position have a limited impact on potency, regardless of their electronic or steric properties.

Disubstituted derivatives also exhibited improved potency, as shown by compounds 12n (2,4-dimethylphenyl) and 12o (2,4-dimethoxyphenyl), with IC_50_ values of 17.07 ± 4.61 µM and 17.28 ± 0.70 µM, respectively.

The trisubstituted derivative compound 12p (3,4,5-trimethoxyphenyl) exhibited weak activity, with % inhibition of 35.14%, suggesting that excessive steric bulk impair binding and inhibitory activity. Replacing the phenyl group with a benzyl one (compound 12q) showed no significant improvement in potency *vs.*12a, indicating that extending the aromatic system does not enhance activity. The same trend were seen in 12r*vs.*12g in which elongation of the linker is not favorable.

Overall, substituents at the 2-position generally improved inhibition, with small electron-withdrawing groups such as fluorine showing the greatest potency. Steric hindrance and electron-donating groups, especially in other positions, tend to reduce activity. These findings underscore the importance of optimizing substituent position for developing potent BChE inhibitors.

### 2.4 Kinetic studies of 12c

The kinetics of BChE inhibition by compound 12c were investigated at various inhibitor concentrations. Different concentrations of the substrates butyrylthiocholine iodide (BTCI) were used to determine initial velocity values. The reciprocal of the substrate concentration (1/[S]) was plotted against the reciprocal of the initial velocity (1/*V*) to generate Lineweaver–Burk plots.

**Fig. 4:**
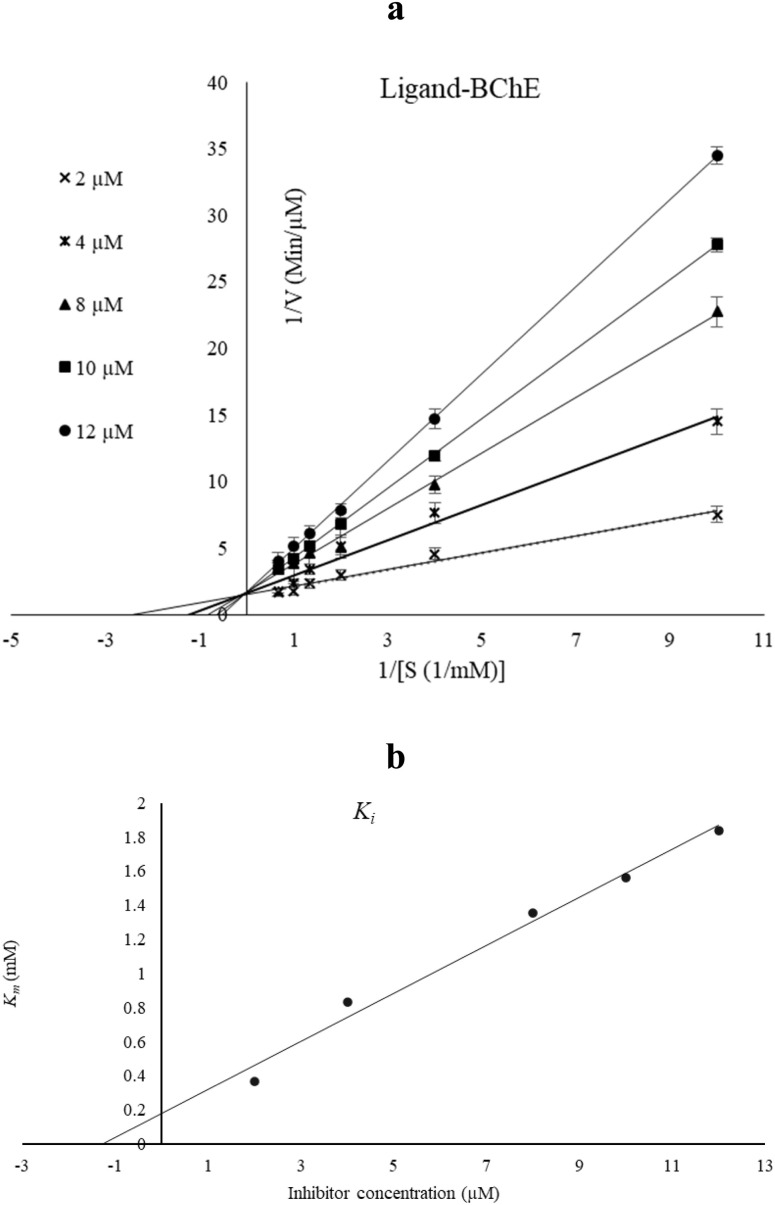
(a) Lineweaver–Burk plot for the inhibition of BChE by 12c in the presence of various concentrations of the substrate. (b) Secondary plot for the calculation of the inhibition constant of compound 12c.

For BChE (Fig. 4a), the Lineweaver–Burk plot showed that both the slope and *y*-intercept increased with increasing inhibitor concentrations, indicating a competitive mode of inhibition. The inhibition constant (*K*_i_) for compound 12c was determined from the secondary plot (Fig. 4b) and found to be 2.05 µM.

### 2.5 Molecular docking studies

Molecular docking studies were conducted to investigate the behavior of the target compound within the AChE and BChE binding sites. First redocking of the crystallographic inhibitor were peromed and RMSD between the docked and experimental poses was found to be less than 2 Å, confirming the reliability of the docking procedure.

The detailed molecular docking (Table 3) and enzyme inhibition data (Table 2) provide clear evidence of the structural features required for effective AChE inhibition. Compounds 12a, 12c, and 12n emerged as the most potent inhibitors with IC_50_ values of 23.06 µM, 7.17 µM, and 6.65 µM, respectively, and docking scores of −14.40, −13.68, and −14.39 kcal mol^−1^. The active molecules show a consistent formation of π–π stacking interactions with Trp86 (located in the choline-binding site) and Trp286 (located in the PAS), as well as significant hydrogen bonding with Glu292, Phe295, and Arg296. The combined interactions seem to be important for achieving high binding affinity and potent inhibitory activity. Compound 12c showed a π-cation interaction with His447, a key residue within the catalytic triad of AChE. This interaction with the catalytic triad is may contribute to superior inhibitory potency compared to other analogs. Compounds 12a and 12n, while still potent, are stabilized mainly by the interactions with the choline-binding site and PAS, without directly targeting the catalytic triad. In contrast, less active or inactive derivatives (such as compounds 12d, 12e, 12f, 12g, 12h and 12i) mostly showed significantly higher docking energies (less favorable binding scores), which correlated with their lower enzyme inhibition percentages.

**Table 3:** Molecular docking study of 12a–s against AChE and BChE

Compound	AChE		BChE	
	Type of interaction with residue	Binding energy	Type of interaction with residue	Binding energy
12a	H-bond Phe295	−14.40	H-bond Ser287	−9.085
	π–π stacking Tyr341		H-bond Thr284	
	H-bond Tyr124		π-cation His438	
	π–π stacking Tyr124		π–π stacking Tyr332	
	H-bond Tyr133			
	π–π stacking Trp86			
	π–π stacking Trp286			
12b	H-bond Glu292	−11.143	H-bond Gly197	−10.804
	H-bond Phe295		2 × π–π stacking Trp82	
	H-bond Arg296		H-bond Pro285	
	π–π stacking Phe337			
	π–π stacking Phe338			
	π–π stacking Tyr341			
	π–π stacking Trp286			
12c	H-bond Glu292	−13.678	H-bond Asp70	−11.443
	H-bond Phe295		H-bond Asn68	
	H-bond Arg296		H-bond Glu197	
	H-bond Tyr124		π–π stacking Tyr332	
	π–π stacking Tyr341		2 × π–π stacking Trp82	
	π–π stacking Phe338			
	π-cation interaction His447			
12d	Halogen bond Asp74	−10.828	Halogen bond Gly197	−10.813
	π–π stacking Tyr341		H-bond Gly197	
	π–π stacking Phe338		π–π stacking Phe329	
	π–π stacking Trp285		H-bond Pro285	
	H-bond Glu292		H-bond Tyr284	
12e	H-bond Tyr124	−9.342	H-bond Thr284	−10.051
	H-bond Leu289		H-bond Asn289	
	π–π stacking Trp86			
	π–π stacking Tyr341			
12f	Halogen bond Gln71	−9.817	Halogen bond Thr122	−9.847
	Halogen bond Val73		π-cation His438	
	π–π stacking Trp286		2 × π–π stacking Tyr332	
			H-bond Ser287	
12g	H-bond Tyr341	−10.858	H-bond Gly116	−9.107
	2 × π–π stacking Trp286		H-bond Ser198	
	π–π stacking Val340		H-bond Ser287	
12h	H-bond Tyr341	−8.212	H-bond Ser72	−9.732
	2 × π–π stacking Tyr341		H-bond Gly116	
	π–π stacking Phe338		π–π stacking Tyr332	
	2 × π–π stacking Trp86		π–π stacking Phe329	
12i	H-bond Tyr124	−9.727	H-bond Asn68	−8.218
	H-bond Asp74		π–π stacking Tyr332	
	π–π stacking Tyr124		2 × π–π stacking Phe329	
	π–π stacking Tyr341			
	π–π stacking Tyr72			
12j	π–π stacking His287	−9.865	π–π stacking Trp82	−9.703
	π–π stacking Tyr124		π–π stacking Phe329	
	π–π stacking Tyr341		π–π stacking Tyr332	
	H-bond Tyr124		H-bond Asn68	
	π–π stacking Tyr337		H-bond Gln119	
12k	π–π stacking Tyr341	−9.410	Halogen bond Asp70	−10.213
	H-bond Trp341		H-bond Tyr128	
	H-bond Phe295		π–π stacking Trp82	
	π–π stacking Tyr337		π–π stacking Tyr332	
12l	Halogen bond Asp74	−9.995	Halogen bond Tyr128	−9.044
	π–π stacking His447		π–π stacking Trp82	
	π–π stacking Tyr124		π–π stacking Tyr332	
	π–π stacking Trp286			
	π–π stacking Trp341			
12m	2 × π–π stacking Tyr341	−9.467	H-bond Asp70	−10.946
	H-bond Tyr124		H-bond Ser72	
			π–π stacking Trp82	
12n	H-bond Glu292	−14.390	H-bond Thr284	−10.169
	H-bond Phe295		H-bond Ser287	
	H-bond Arg296		π-cation His438	
	π–π stacking Tyr337			
	π–π stacking Tyr341			
	π–π stacking Trp86			
12o	2 × H-bond Tyr124	−10.820	H-bond Ser287	−9.085
	π–π stacking Tyr124		H-bond Thr284	
	π–π stacking Tyr341		π-cation His438	
	π–π stacking Trp86		π–π stacking Tyr332	
	π–π stacking Trp286			
12p	2 × π–π stacking Tyr341	−7.138	H-bond Gly197	−10.804
	π–π stacking Phe338		2 × π–π stacking Trp82	
			H-bond Pro285	
12q	π–π stacking Trp286	−12.514	H-bond Asp70	−11.443
	2 × H-bond Glu292		H-bond Asn68	
	π–π stacking Tyr341		H-bond Glu197	
	π–π stacking Tyr337		π–π stacking Tyr332	
			2 × π–π stacking Trp82	
12r	π–π stacking Trp86	−12.514	H-bond Thr230	−9.443
	H-bond Asp74		H-bond Tyr128	
	H-bond Gly202			
	H-bond Phe295			
12s	H-bond Gly202	−9.245	π–π stacking Tyr82	−8.085
	π–π stacking Trp286		H-bond Ser287	
	π–π stacking Tyr341			

The comparative examination of BChE docking scores and molecular interactions reveals striking features accounting for significant increases in activity. The most potent derivatives seem to include 12c (IC_50_ = 4.37 µM, binding energy: −11.443 kcal mol^−1^), 12b (IC_50_ = 10.07 µM, −10.804 kcal mol^−1^), 12n (IC_50_ = 17.07 µM, −10.169 kcal mol^−1^), and 12o (IC_50_ = 17.28 µM, −10.685 kcal mol^−1^), as they showed much stronger docking scores than the less active analogs who had mostly binding energies between −9.0 and −10.2 kcal mol^−1^. Most importantly, these potent compounds appeared to form multiple hydrogen bonds with critical residues of Gly197, Glu197, Asn68, Pro285, and Thr284, and Ser287. Interestingly, the hydrogen bonding with Gly197 was common with active compounds 12b and 12d, but absent or much less frequent in the less active analogs. Moreover, 12c was unique in exhibiting hydrogen bonding with Glu197 and Asn68, interactions not observed in weaker derivatives, indicating that these polar contacts are responsible for 12c superior binding affinity. Also, Pro285 contributed to the stabilization of binding for the potent compounds but showed little to no involvement in interactions with the less active compounds. Whereas π–π stacking with Trp82 and Tyr332 was observed in both weak and potent variants, its contribution appears most significant when combined with other interactions, especially hydrogen bonding. Altogether, hydrogen bonding with Gly197, Glu197, Asn68, Pro285, and π–π stacking with Trp82 seems favorable.

**Fig. 5:**
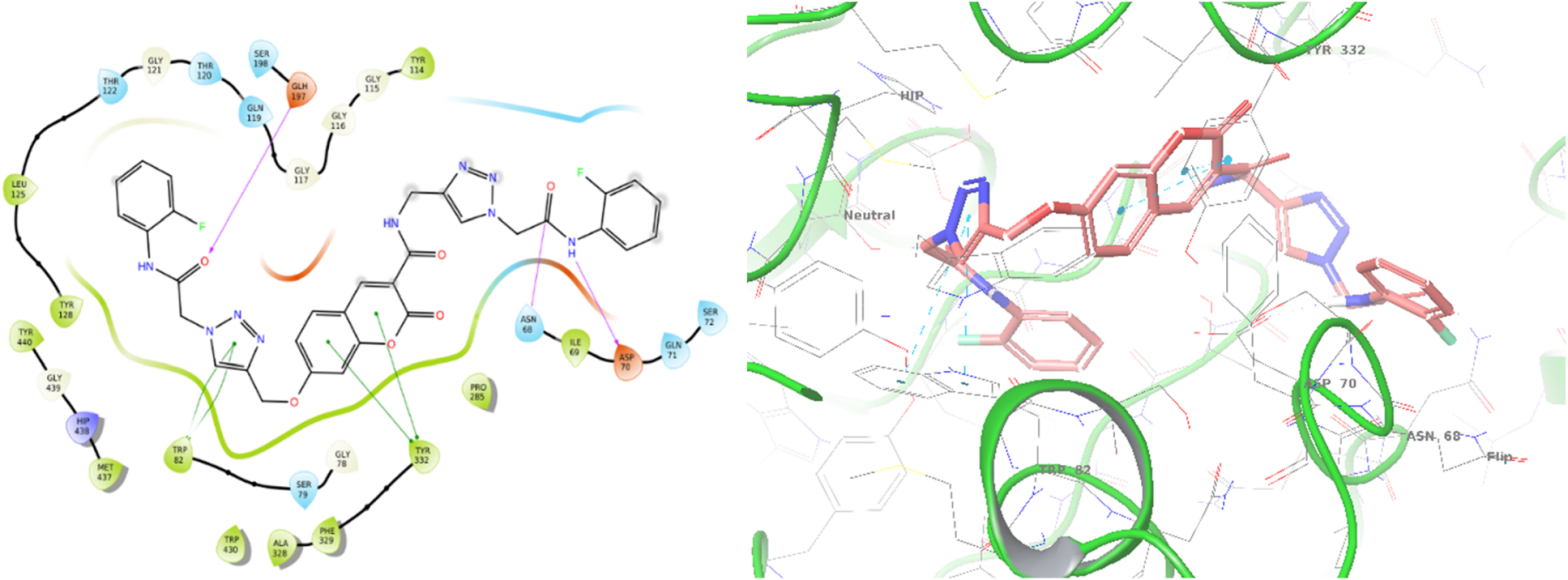
Proposed binding mode for compound 12c docked at the active site of BChE.

The top-ranked pose of compound 12c as a BChE inhibitor within the binding site is illustrated in Fig. 5.

**Fig. 6:**
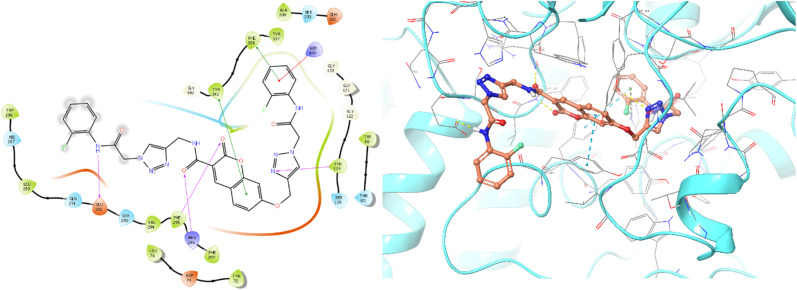
Proposed binding mode for compound 12c docked at the active site of AChE.

The top-ranked pose of compound 12c as an AChE inhibitor within the binding site is illustrated in Fig. 6.

### 2.6 Molecular dynamics simulations

**Fig. 7:**
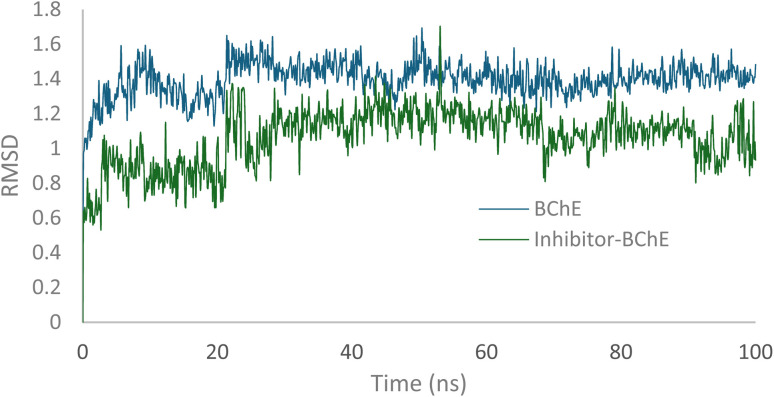
RMSD of BChE *vs.*12c-BChE.

To examine the binding stability, MD simulations were run for the 12c-BChE complex. As shown in Fig. 7, the inhibited BChE exhibited slight structural variation and reached equilibrium after approximately 35 ns. The average RMSD value for the compound binding stability within the enzyme's active site was 1.0 Å. In comparison, BChE alone achieved stability after 33 ns with an average RMSD of 1.4 Å. These findings confirm that the overall folding of the ternary complex remained essentially stable throughout the simulation.

**Fig. 8:**
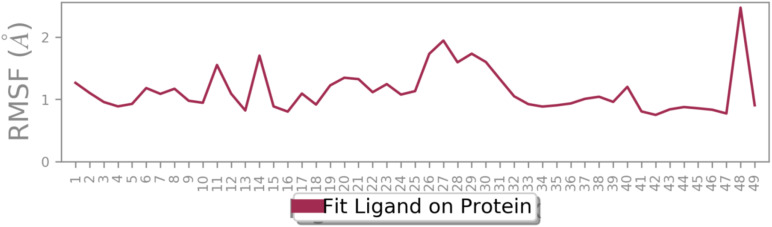
RMSD of 12c in the active site of BChE.

Following this trend, the ligand exhibited minimal fluctuations within the enzyme's binding site, as indicated by RMSF values. All atoms showed RMSF values below 2 Å, except for number 48, which displayed a slightly higher RMSF of 2.3 Å. These data confirm the stability of the ligand within the active site of BChE (Fig. 8).

Based on the outcomes of molecular docking and MD simulation, it was determined that all 12c moieties participated in interactions with various binding pockets of the enzyme. Analyzing the interactions between the ligand and amino acid residues in the protein's active site is a crucial part of assessing the stability of the protein–ligand complex. The analysis of the full MD simulation run (0–100 ns) showed that the complex formed multiple stable interactions during the simulation, the amide linker attached to the 2-fluorophenyl formed hydrogen bond with Asp70 of the peripheral anionic site which facilitated interactions with the entrance of the enzyme's binding site, and the triazole moiety attached to this linker interacted with Ser287.

**Fig. 9:**
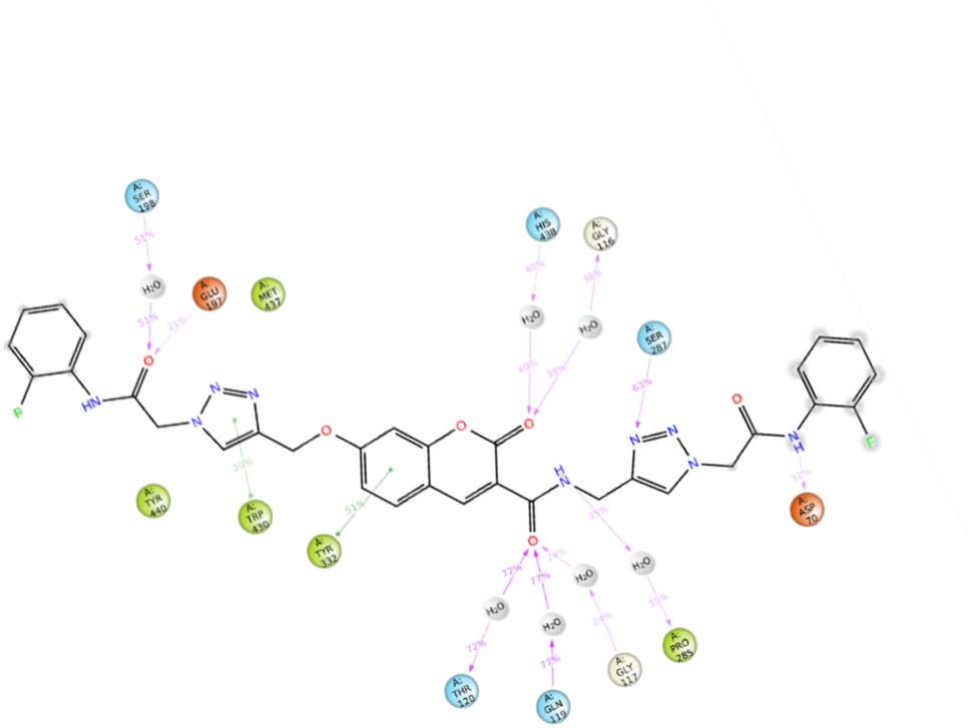
A schematic of detailed 12c atom interactions with the protein residues. Interactions that occur more than 20.0% of the simulation time in the selected trajectory.

Also, the amide linker attached to the chromenone core formed several hydrogen-bond interactions with Pro285, mediated by a water molecule and residues in the oxanion hole region, such as Gly117, Gln119, and Thr120. The MD simulation demonstrates that water molecules mediate the stabilization of the complex, as underscored by these water-bridged interactions (Fig. 9).

Additionally, the chromenone core established two hydrogen bonds, both mediated by water molecules: one with Gly116 (the oxanion hole area) and another with His438 (a member of the catalytic triad). Two hydrogen bonds involving the amide linker connected to the terminal 2-fluorophenyl group were established with Glu197 and Ser198 (catalytic triad). The current docking study repeatedly found this pattern of interaction. The methoxy triazole linker contributed to binding stability by participating in π–π stacking interactions with Trp430.

**Fig. 10:**
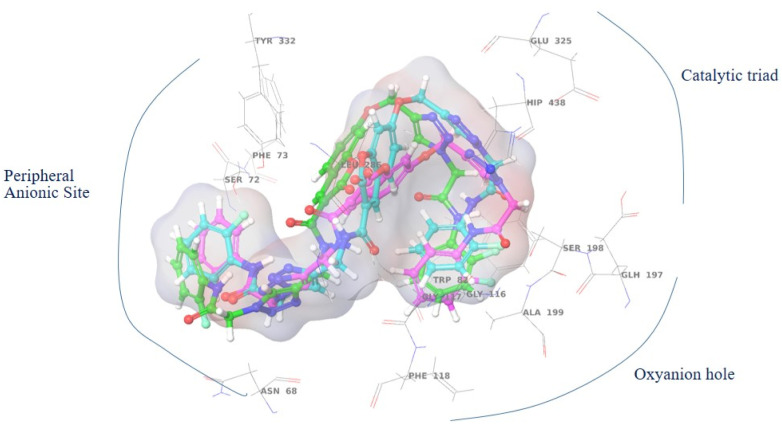
The conformation of 12c over different time trajectories during MD simulation.

The compound's 3D interaction behavior within the BChE binding pocket was assessed at the start, middle, and end of the simulation to verify its stability and occupation over time. The results showed that the compound adopted an S-shaped conformation during the MD run, confirming that various moieties of the molecule successfully occupied distinct pockets of the enzyme during the simulation (Fig. 10). Based on the outcomes of molecular docking and MD simulation, it was determined that all 12c moieties participated in interactions with various binding pockets of the enzyme.

**Fig. 11:**
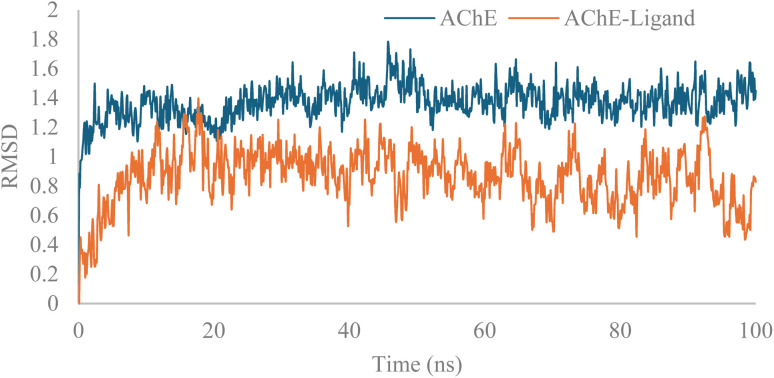
RMSD of BChE *vs.*12c-AChE.

Molecular dynamics simulation of 12c in the AChE active site was also performed, and the RMSD of the 12c-AChE *vs.* AChE complexes are shown in Fig. 11. The apo enzyme and the ligand-bound complex displayed structural stability within the 100 ns simulation. The apo AChE had an average RMSD of approximately 1.5 Å, whereas the 12c-AChE complex showed a lower RMSD value of approximately 0.9 Å. This reduction of RMSD upon ligand binding suggests that compound 12c facilitates the stabilization of the structure of AChE during the simulation (Fig. 11).

**Fig. 12:**
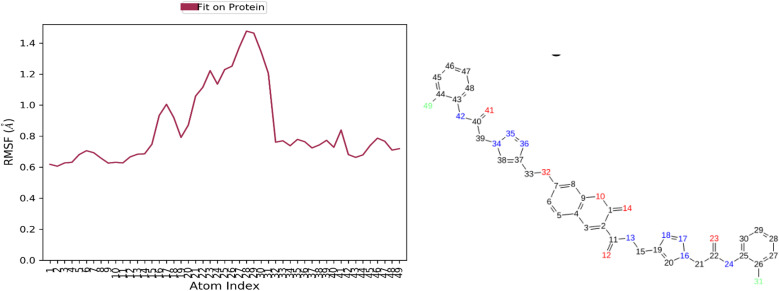
RMSD of 12c in the active site of AChE.

Additionally, all atoms of the ligand exhibited RMSF values below 2 Å, indicating minimal atomic fluctuations during the simulation. These results confirm the structural stability of the ligand within the active site of AChE (Fig. 12).

**Fig. 13:**
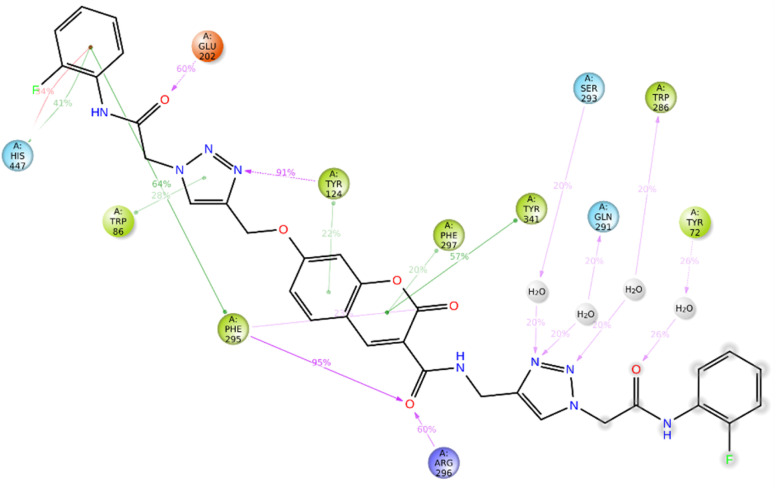
A schematic of detailed 12c atom interactions with the protein residues. Interactions that occur more than 20.0% of the simulation time in the selected trajectory.

12c binding interactions with AChE were also investigated. One of the 2-fluorophenyl groups exhibited both π-cation and π–π stacking interactions with His447, a crucial residue of the catalytic triad. The amide linker among the 2-fluorophenyl ring formed hydrogen bonds with Tyr72 (*via* a water molecule) and Glu202. The 1,2,4-triazole ring was involved in direct hydrogen bonding with Tyr124, π–π stacking with Trp86, and three water-assisted hydrogen bonds with Ser293, Glu291, and Trp286. The chromenone core was also involved in some stabilizing interactions like π–π stacking with Tyr124, Phe297, and Tyr341, and one hydrogen bond with Phe295 (Fig. 13).

### 2.7 Cell cytotoxicity

**Fig. 14:**
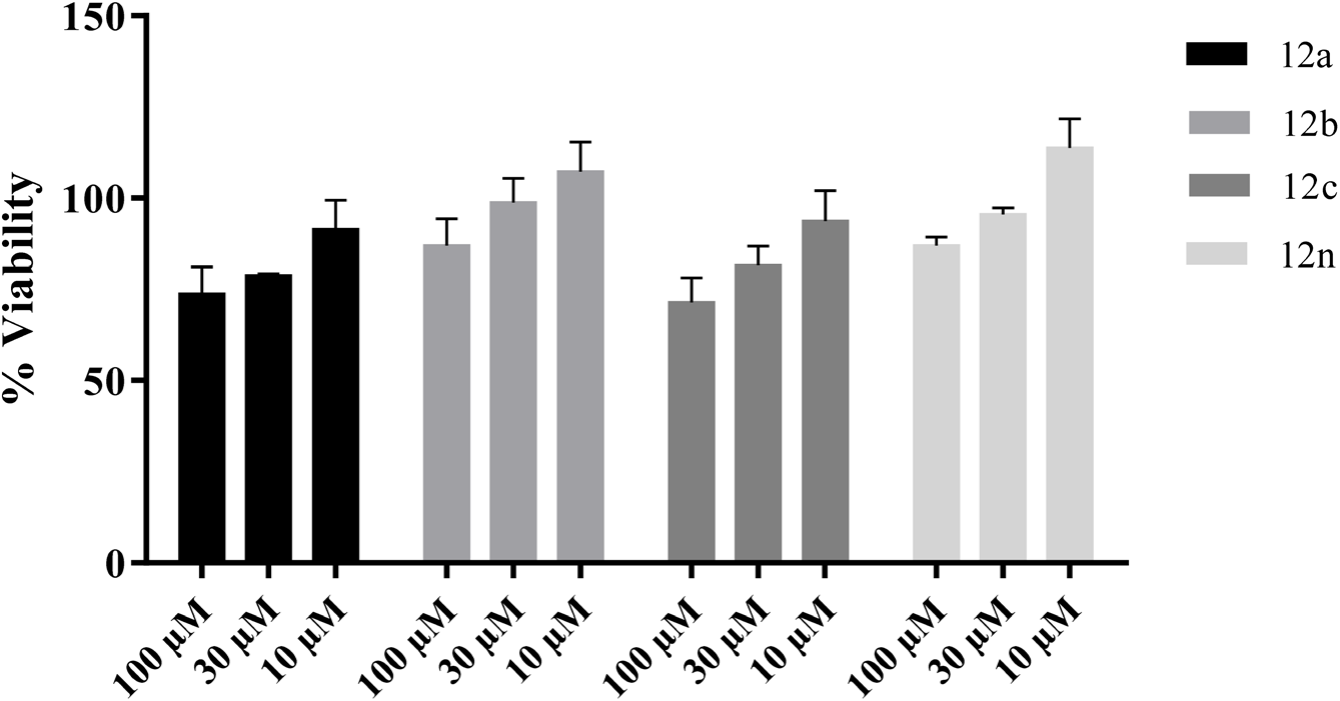
Cytotoxicity study of 12a, 12b, 12c and 12r against the SH-SY5Y.

The most active derivatives were tested for cytotoxicity in the SH-SY5Y neuroblastoma cell line (Pasteur Institute of Iran (https://en.pasteur.ac.ir/)) to estimate their neurotoxicity. Cell viability was assessed 72 h after treatment, and the results are summarized in Fig. 14. The data indicate that all compounds showed no significant cytotoxicity at 10 µM and maintained >80% cell viability at 30 µM. At 10 µM, all derivatives showed cell viability greater than 70%. Given the IC_50_ value of compound 12c, this analogue can be considered relatively safe under the tested conditions.

### 2.8 Prediction of pharmacokinetic properties

These physicochemical attributes are essential for developing effective treatments for neurological disorders.^23^ As summarized in Table 4, all selected compounds exhibit molecular weights ranging from 633.62 to 689.73 Da. The number of rotatable bonds is consistent across the series (RB = 12), indicating a comparable degree of molecular flexibility. The calculated topological polar surface area (tPSA) values range from 265.70 to 291.16 Å^2^, reflecting the relatively polar nature of these structures. All compounds contain 12 hydrogen-bond acceptors and 3 hydrogen-bond donors, suggesting similar hydrogen-bonding capacities across the series. The calculated Log *P* values (2.76–3.99) indicate moderate lipophilicity, remaining within commonly accepted limits for oral drug candidates.

**Table 4:** Physicochemical properties of the synthesized compounds 12a, 12b, 12c and 12n

Compound	*M*_W_ (Da)	log *P*	HBA	HBD	tPSA (Å^2^)	RB
12a	633.625	2.7624	12	3	265.701	12
12b	661.680	3.3792	12	3	278.431	12
12c	669.605	3.0406	12	3	274.032	12
12n	689.733	3.9960	12	3	291.161	12

ADME predictions (Table 5) revealed moderate to high predicted intestinal absorption (67.60–72.32%), suggesting acceptable gastrointestinal uptake. All compounds were predicted to be P-glycoprotein (P-gp) substrates and CYP3A4 substrates, and to act as CYP3A4 inhibitors, indicating potential susceptibility to efflux transport and drug–drug interactions. None of the compounds were predicted to inhibit CYP2C19 or CYP1A2, and none were predicted to act as CYP2D6 substrates.

**Table 5:** ADMEa prediction of the synthesized compounds 12a, 12b, 12c and 12n

Compound	VDss (human)	Intestinal absorption (%)	P-gp substrate	CYP3A4 inhibitor	CYP2C19 inhibitor	CYP1A2 inhibitor	CYP2D6 substrate	CYP3A4 substrate
12a	−0.727	71.38	Yes	Yes	No	No	No	Yes
12b	−0.694	72.316	Yes	Yes	No	No	No	Yes
12c	−0.821	67.605	Yes	Yes	No	No	No	Yes
12n	−0.66	73.253	Yes	Yes	No	No	No	Yes

## 3. Conclusion

In this study, a series of triazole hybrids (12a–s) were successfully synthesized and their inhibitory activities against BChE and AChE enzymes were evaluated. The results showed that structural modifications, particularly the positions of substituents, significantly affect biological activity. In terms of BChE inhibition, compounds with 2-position substituents, particularly 2-fluorophenyl (compound 12c, IC_50_ = 4.37 ± 0.91 µM), generally increased potency, while compounds with 2-position substituents showed the second-highest activity. As demonstrated by compound 12n (2,4-dimethylphenyl; IC_50_ = 6.65 ± 1.31 µM), disubstitution at the 2,4-positions enhanced binding affinity, highlighting the advantageous effects of dual substitutions. Overall, the study showed that although steric hindrance and excessive bulk reduce activity, small electron-withdrawing groups, especially fluorine are quite effective at boosting enzyme inhibition. Through docking assessments, compound 12c showed a substantial binding affinity to both AChE and BChE. The stability of the 12c-enzyme complexes was validated by MD over 100 ns, which showed persistent hydrogen bonding and low RMSD values.

These findings provide valuable insights into the structure–activity relationships of triazole hybrids and establish a foundation for further optimization and development of potent cholinesterase inhibitors for therapeutic applications in Alzheimer's disease.

## 4. Method and materials

### 4.1 Chemistry

#### 4.1.1 2-oxo-7-((1-(2-oxo-2-(Phenylamino)ethyl)-1*H*-1,2,3-triazol-4-yl)methoxy)-*N*-((1-(2-oxo-2-(phenylamino)ethyl)-1*H*-1,2,3-triazol-4-yl)methyl)-2*H*-chromene-3-carboxamide (12a)

Brown solid; yield: 67%; MP = 212–214 °C; IR (KBr, *v*_max_) 3310 (NH), 3016 (CH aromatic), 2945 (CH aliphatic), 1662 (C=O) cm^−1^; ^1^H NMR (500 MHz, DMSO-*d*_6_) *δ* 10.48 (s, 1H), 10.44 (s, 1H), 9.10 (t, *J* = 4.8 Hz, 1H), 8.87 (s, 1H), 8.33 (s, 1H), 8.05 (s, 1H), 7.93 (d, *J* = 8.7 Hz, 1H), 7.62–7.50 (m, 4H), 7.37–7.26 (m, 5H), 7.13 (d, *J* = 8.4 Hz, 1H), 7.10–7.04 (m, 2H), 5.37–5.34 (m, 4H), 5.31 (s, 2H), 4.62 (d, *J* = 4.5 Hz, 2H): ^13^C NMR (126 MHz, DMSO-*d*_6_) *δ* 164.16, 164.08, 163.14, 161.39, 160.78, 156.07, 147.94, 138.37, 138.34, 131.62, 128.85, 126.68, 124.59, 123.76, 123.73, 119.23, 114.84, 114.08, 112.33, 101.15, 61.90, 52.23, 52.19, 34.80; anal. calcd; C_32_H_27_N_9_O_6_; C, 60.66; H, 4.30; N, 19.90; found; C, 60.82; H, 4.48; N, 20.08.

#### 4.1.2 2-oxo-7-((1-(2-oxo-2-(*o*-Tolylamino)ethyl)-1*H*-1,2,3-triazol-4-yl)methoxy)-*N*-((1-(2-oxo-2-(*o*-tolylamino)ethyl)-1*H*-1,2,3-triazol-4-yl)methyl)-2*H*-chromene-3-carboxamide (12b)

Brown solid; yield: 78%; MP = 222–225 °C; IR (KBr, *v*_max_) 3315 (NH), 3025 (CH aromatic), 2850 (CH aliphatic), 1663 (C=O) cm^−1^; ^1^H NMR (500 MHz, DMSO-*d*_6_) *δ* 9.80 (s, 1H), 9.76 (s, 1H), 9.10 (t, *J* = 5.5 Hz, 1H), 8.87 (s, 1H), 8.33 (s, 1H), 8.05 (s, 1H), 7.93 (d, *J* = 8.8 Hz, 1H), 7.41 (d, *J* = 7.8 Hz, 2H), 7.31 (d, *J* = 1.9 Hz, 1H), 7.22 (d, *J* = 7.2 Hz, 2H), 7.18–7.12 (m, 3H), 7.12–7.07 (m, 3H), 5.41 (s, 2H), 5.36 (s, 2H), 5.34 (s, 2H), 4.61 (d, *J* = 5.2 Hz, 2H), 2.23–2.21 (m, 6H): ^13^C NMR (126 MHz, DMSO-*d*_6_) *δ* 164.86, 164.78, 163.64, 161.88, 161.28, 156.58, 148.45, 144.61, 141.98, 135.99, 135.96, 132.12, 132.06, 130.90, 127.17, 126.52, 126.04, 126.01, 125.20, 125.03, 115.34, 114.59, 112.83, 101.64, 62.39, 52.45, 52.41, 35.30, 18.23; anal. calcd; C_34_H_31_N_9_O_6_; C, 61.72; H, 4.72; N, 19.05; found; C, 61.91; H, 4.87; N, 19.28.

#### 4.1.3 7-((1-(2-((2-Fluorophenyl)amino)-2-oxoethyl)-1*H*-1,2,3-triazol-4-yl)methoxy)-*N*-((1-(2-((2-fluorophenyl)amino)-2-oxoethyl)-1*H*-1,2,3-triazol-4-yl)methyl)-2-oxo-2*H*-chromene-3-carboxamide (12c)

Brown solid; yield: 65%; MP = 230–232 °C; IR (KBr, *v*_max_) 3350 (NH), 3040 (C–H aromatic), 2960 (CH aliphatic), 1667 (C=O) cm^−1^; ^1^H NMR (500 MHz, DMSO-*d*_6_) *δ* 10.33 (s, 1H), 10.29 (s, 1H), 9.10 (t, *J* = 5.2 Hz, 1H), 8.88 (s, 1H), 8.80 (s, 1H), 8.67 (d, *J* = 14.2 Hz, 1H), 8.32 (s, 1H), 8.04 (s, 1H), 7.94 (d, *J* = 8.7 Hz, 1H), 7.92–7.86 (m, 2H), 7.33–7.24 (m, 2H), 7.22–7.08 (m, 4H), 5.45 (s, 2H), 5.40 (s, 2H), 5.35 (s, 2H), 4.61 (d, *J* = 5.1 Hz, 2H): ^13^C NMR (126 MHz, DMSO-*d*_6_) *δ* 164.84, 164.80, 163.18, 161.61., 160.90, 157.57, 156.18, 153.54 (d, *J* = 246.0 Hz), 148.09, 141.73, 135.94, 131.74, 130.76, 126.85, 125.77, 125.55, 124.54, 123.81, 120.37, 115.75, 115.60, 114.20, 11.85, 101.59, 101.21, 61.97, 52.07, 52.04, 34.90; anal. calcd; C_32_H_25_F_2_N_9_O_6_; C, 57.40; H, 3.76; N, 18.83; found; C, 57.56; H, 3.91; N, 19.02. The purity of the compound was checked using HPLC (98.2% purity).

#### 4.1.4 7-((1-(2-((2-Chlorophenyl)amino)-2-oxoethyl)-1*H*-1,2,3-triazol-4-yl)methoxy)-*N*-((1-(2-((2-chlorophenyl)amino)-2-oxoethyl)-1*H*-1,2,3-triazol-4-yl)methyl)-2-oxo-2*H*-chromene-3-carboxamide (12d)

Cream solid; yield: 61%; MP = 239–241 °C; IR (KBr, *v*_max_) 3330 (NH), 3040 (CH aromatic), 2960 (CH aliphatic), 1662 (C=O) cm^−1^; ^1^H NMR (500 MHz, DMSO-*d*_6_) *δ* 10.09 (s, 1H), 10.04 (s, 1H), 9.10 (t, *J* = 4.8 Hz, 1H), 8.87 (s, 1H), 8.33 (s, 1H), 8.05 (s, 1H), 7.93 (d, *J* = 8.7 Hz, 1H), 7.72 (d, *J* = 8.1 Hz, 2H), 7.51 (d, *J* = 9.0 Hz, 2H), 7.37–7.29 (m, 3H), 7.27–7.17 (m, 2H), 7.12 (d, *J* = 10.8 Hz, 1H), 5.47 (s, 2H), 5.41 (s, 2H), 5.35 (s, 2H), 4.61 (d, *J* = 4.6 Hz, 2H): ^13^C NMR (126 MHz, DMSO-*d*_6_) *δ* 165.01, 164.93, 163.23, 161.50, 160.90, 148.09, 134.23, 134.21, 131.74, 129.69, 127.63, 126.85, 126.38, 126.11, 125.78, 124.78, 114.91, 114.21, 112.43, 101.23, 61.96, 52.07, 52.01, 34.92; anal. calcd; C_32_H_25_C_12_N_9_O_6_; C, 54.71; H, 3.59; N, 17.94; found; C, 54.93; H, 3.76; N, 18.14.

#### 4.1.5 2-oxo-7-((1-(2-oxo-2-(*m*-Tolylamino)ethyl)-1*H*-1,2,3-triazol-4-yl)methoxy)-*N*-((1-(2-oxo-2-(*m*-tolylamino)ethyl)-1*H*-1,2,3-triazol-4-yl)methyl)-2*H*-chromene-3-carboxamide (12e)

Brown solid; yield: 71%; MP = 251–253 °C; IR (KBr, *v*_max_) 3325 (NH), 3049 (C–H aromatic), 2963 (CH aliphatic), 1665 (C=O) cm^−1^; ^1^H NMR (500 MHz, DMSO-*d*_6_) *δ* 10.67–10.27 (m, 2H), 9.11 (s, 1H), 8.88 (s, 1H), 8.33 (s, 1H), 8.04 (s, 1H), 7.93 (t, *J* = 5.8 Hz, 1H), 7.40 (s, 1H), 7.38–7.33 (m, 2H), 7.31 (s, 1H), 7.27–7.16 (m, 3H), 7.13 (d, *J* = 9.0 Hz, 1H), 6.99–6.75 (m, 2H), 5.35 (m, 4H), 5.30 (s, 2H), 4.61 (d, *J* = 5.7 Hz, 2H), 2.41–2.11 (m, 6H); ^13^C NMR (126 MHz, DMSO-*d*_6_) *δ* 164.15, 164.11, 164.06, 163.15, 161.42, 160.84, 156.11, 148.03, 141.45, 138.33, 138.13, 131.67, 128.74, 126.79, 124.49, 124.46, 119.75, 116.39, 114.81, 114.12, 112.35, 101.12, 61.88, 52.26, 52.20, 34.82, 21.15; anal. calcd; C_34_H_31_N_9_O_6_; C, 61.72; H, 4.72; N, 19.05; found; C, 61.90; H, 4.91; N, 19.25.

#### 4.1.6 7-((1-(2-((3-Chlorophenyl)amino)-2-oxoethyl)-1*H*-1,2,3-triazol-4-yl)methoxy)-*N*-((1-(2-((3-chlorophenyl)amino)-2-oxoethyl)-1*H*-1,2,3-triazol-4-yl)methyl)-2-oxo-2*H*-chromene-3-carboxamide (12f)

Cream solid; yield: 63%; MP = 235–237 °C; IR (KBr, *v*_max_) 3320 (NH), 3040 (CH aromatic), 2950 (CH aliphatic), 1665 (C=O) cm^−1^, ^1^H NMR (500 MHz, DMSO-*d*_6_) *δ* 10.09 (s, 1H), 10.04 (s, 1H), 9.10 (t, *J* = 5.1 Hz, 1H), 8.87 (s, 1H), 8.33 (s, 1H), 8.05 (s, 1H), 7.93 (d, *J* = 8.7 Hz, 1H), 7.85 (d, *J* = 8.5 Hz, 1H), 7.73 (d, *J* = 7.7 Hz, 2H), 7.55–7.47 (m, 2H), 7.38–7.29 (m, 2H), 7.27–7.17 (m, 2H), 7.13 (d, *J* = 8.8 Hz, 1H), 5.47 (s, 2H), 5.42 (s, 2H), 5.35 (s, 2H), 4.61 (d, *J* = 4.7 Hz, 2H): ^13^C NMR (126 MHz, DMSO-*d*_6_) *δ* 165.00, 164.93, 163.23, 161.51, 160.90, 156.19, 148.09, 142.07, 141.64, 136.03, 134.24, 134.22, 131.75, 130.74, 129.71, 129.69, 127.64, 126.83, 126.80, 126.41, 126.30, 125.97, 125.93, 124.81, 124.72, 114.91, 114.21, 112.44, 111.81, 101.24, 61.96, 59.83, 52.06, 52.02, 34.92, 20.83, 14.16; anal. calcd; C_32_H_25_C_12_N_9_O_6_; C, 54.71; H, 3.59; N, 17.94; found; C, 54.92; H, 3.77; N, 18.15.

#### 4.1.7 2-oxo-7-((1-(2-oxo-2-(*p*-Tolylamino)ethyl)-1*H*-1,2,3-triazol-4-yl)methoxy)-*N*-((1-(2-oxo-2-(*p*-tolylamino)ethyl)-1*H*-1,2,3-triazol-4-yl)methyl)-2*H*-chromene-3-carboxamide (12g)

Brown solid; yield: 79%; MP = 220–223 °C; IR (KBr, *v*_max_) 3330 (NH), 3035 (CH aromatic), 2850 (CH aliphatic), 1666 (C=O) cm^−1^, ^1^H NMR (500 MHz, DMSO-*d*_6_) *δ* 10.35 (s, 1H), 10.32 (s, 1H), 9.08 (t, *J* = 4.9 Hz, 1H), 8.87 (s, 1H), 8.31 (s, 1H), 8.03 (s, 1H), 7.93 (d, *J* = 8.7 Hz, 1H), 7.48–7.42 (m, 4H), 7.30 (s, 1H), 7.16–7.05 (m, 5H), 5.35 (s, 2H), 5.33 (s, 2H), 5.27 (s, 2H), 4.61 (d, *J* = 5.1 Hz, 2H), 2.24 (s, 6H): ^13^C NMR (126 MHz, DMSO-*d*_6_) *δ* 164.39, 164.30, 163.58, 161.87, 161.27, 156.58, 148.51, 146.10, 141.96, 136.36, 136.34, 133.25, 132.13, 132.11, 129.72, 129.71, 129.70, 127.21, 127.18, 125.07, 119.76, 115.29, 114.54, 112.83, 101.64, 62.30, 52.71, 52.70, 35.32, 20.87; anal. calcd; C_34_H_31_N_9_O_6_; C, 61.72; H, 4.72; N, 19.05; found; C, 62.12; H, 5.15; N, 19.41. HRMS (TOF MS E+) *m*/*z* calcd for C_34_H_31_N_9_O_6_Na [M + Na]^+^ 684.6697; found 684.3505.

#### 4.1.8 7-((1-(2-((4-Ethylphenyl)amino)-2-oxoethyl)-1*H*-1,2,3-triazol-4-yl)methoxy)-*N*-((1-(2-((4-ethylphenyl)amino)-2-oxoethyl)-1*H*-1,2,3-triazol-4-yl)methyl)-2-oxo-2*H*-chromene-3-carboxamide (12h)

Cream solid; yield: 68%; MP = 212–214 °C; IR (KBr, *v*_max_) 3230 (NH), 3025 (CH aromatic), 2945 (CH aliphatic), 1663 (C=O) cm^−1^, ^1^H NMR (500 MHz, DMSO-*d*_6_) *δ* 10.40 (s, 1H), 10.37 (s, 1H), 9.10 (t, *J* = 5.0 Hz, 1H), 8.88 (s, 1H), 8.32 (s, 1H), 8.04 (s, 1H), 7.93 (d, *J* = 8.8 Hz, 1H), 7.47 (d, *J* = 8.1 Hz, 4H), 7.31 (s, 1H), 7.20–7.09 (m, 5H), 5.36–5.33 (m, 4H), 5.28 (s, 2H), 4.61 (d, *J* = 5.2 Hz, 2H), 2.54 (q, *J* = 7.2 Hz, 4H), 1.14 (t, *J* = 7.4 Hz, 6H); ^13^C NMR (126 MHz, DMSO-*d*_6_) *δ* 164.05, 163.97, 163.26, 161.52, 160.93, 156.20, 148.10, 141.56, 139.29, 136.18, 131.75, 128.17, 126.86, 119.41, 114.91, 114.22, 112.45, 101.24, 61.99, 52.32, 52.27, 34.93, 27.66, 15.67; anal. calcd; C_36_H_35_N_9_O_6_; C, 62.69; H, 5.12; N, 18.28; found; C, 62.85; H, 5.30; N, 18.44.

#### 4.1.9 7-((1-(2-((4-Methoxyphenyl)amino)-2-oxoethyl)-1*H*-1,2,3-triazol-4-yl)methoxy)-*N*-((1-(2-((4-methoxyphenyl)amino)-2-oxoethyl)-1*H*-1,2,3-triazol-4-yl)methyl)-2-oxo-2*H*-chromene-3-carboxamide (12i)

Brown solid; yield: 77%; MP = 253–255 °C; IR (KBr, *v*_max_) 3328 (NH), 3042 (C–H aromatic), 2964 (CH aliphatic), 1663 (C=O) cm^−1^; 1H NMR (400 MHz, DMSO-*d*_6_) *δ* 10.57–10.20 (m, 2H), 9.13 (t, *J* = 5.8 Hz, 1H), 8.90 (s, 1H), 8.35 (s, 1H), 8.06 (s, 1H), 7.96 (d, *J* = 8.6 Hz, 1H), 7.50 (d, *J* = 8.6 Hz, 4H), 7.34 (d, *J* = 2.4 Hz, 1H), 7.15 (dd, *J* = 8.7, 2.4 Hz, 1H), 6.97–6.85 (m, 4H), 5.40–5.33 (m, 4H), 5.29 (s, 2H), 4.63 (d, *J* = 5.6 Hz, 2H), 3.72 (s, 6H); ^13^C NMR (101 MHz, DMSO-*d*_6_) *δ* 164.16, 164.08, 163.62, 163.59, 162.11, 161.89, 161.31, 156.59, 155.96, 148.51, 132.16, 131.98, 131.95, 127.28, 121.21, 121.20, 114.61, 114.47, 114.42, 55.61, 52.59, 52.58, 42.99; anal. calcd; C_34_H_31_N_9_O_8_; C, 58.87; H, 4.50; N, 18.17; found; C, 59.03; H, 4.68; N, 18.37.

#### 4.1.10 7-((1-(2-((4-Fluorophenyl)amino)-2-oxoethyl)-1*H*-1,2,3-triazol-4-yl)methoxy)-*N*-((1-(2-((4-fluorophenyl)amino)-2-oxoethyl)-1*H*-1,2,3-triazol-4-yl)methyl)-2-oxo-2*H*-chromene-3-carboxamide (12j)

Brown solid; yield: 74%; MP = 239–241 °C; IR (KBr, *v*_max_) 3350 (NH), 3025 (C–H aromatic), 2980 (CH aliphatic), 1669 (C=O) cm^−1^; ^1^H NMR (500 MHz, DMSO-*d*_6_) *δ* 10.56 (s, 1H), 10.52 (s, 1H), 9.10 (t, *J* = 4.7 Hz, 1H), 8.87 (s, 1H), 8.33 (s, 1H), 8.05 (s, 1H), 7.93 (d, *J* = 8.7 Hz, 1H), 7.68–7.51 (m, 4H), 7.31 (s, 1H), 7.24–7.12 (m, 5H), 5.38–5.33 (m, 4H), 5.30 (s, 2H), 4.61 (d, *J* = 4.7 Hz, 2H): ^13^C NMR (126 MHz, DMSO-*d*_6_) *δ* 164.25, 164.17, 163.23, 161.51, 160.91, 158.34 (d, *J* = 238.5 Hz), 156.17, 148.08, 141.62, 134.85, 134.84, 134.82, 131.72, 126.84, 124.74, 121.14 (d, *J* = 7.8 Hz), 115.56 (d, *J* = 22.3 Hz), 114.88, 114.19, 112.43, 101.22, 61.98, 52.27, 52.23, 34.92; ^19^F NMR (471 MHz, DMSO-*d*_6_) *δ* −118.65 (dtt, *J* = 21.3, 8.7, 4.8 Hz); anal. calcd; C_32_H_25_F_2_N_9_O_6_; C, 57.40; H, 3.76; N, 18.83; found; C, 57.62; H, 3.91; N, 18.98.

#### 4.1.11 7-((1-(2-((4-Chlorophenyl)amino)-2-oxoethyl)-1*H*-1,2,3-triazol-4-yl)methoxy)-*N*-((1-(2-((4-chlorophenyl)amino)-2-oxoethyl)-1*H*-1,2,3-triazol-4-yl)methyl)-2-oxo-2*H*-chromene-3-carboxamide (12k)

Brown solid; yield: 68%; MP = 240–242 °C; IR (KBr, *v*_max_) 3320 (NH), 3030 (CH aromatic), 2940 (CH aliphatic),1662 (C=O) cm^−1^; ^1^H NMR (500 MHz, DMSO-*d*_6_) *δ* 10.62 (s, 1H, NH), 10.59 (s, 1H, NH), 9.10 (t, *J* = 5.5 Hz, 1H, H13), 8.88 (s, 1H, triazole), 8.33 (s, 1H, triazole), 8.04 (s, 1H, H6), 7.94 (d, *J* = 8.8 Hz, 1H, H6), 7.59 (d, *J* = 8.1 Hz, 4H), 7.40–7.36 (m, 4H), 7.31 (d, *J* = 2.0 Hz, 1H, H3), 7.13 (dd, *J* = 8.7, 2.2 Hz, 1H, H1), 5.37 (s, 2H, CH_2_), 5.35 (s, 2H, CH_2_), 5.31 (s, 2H, CH_2_), 4.61 (d, *J* = 5.3 Hz, 2H, H15): ^13^C NMR (126 MHz, DMSO-*d*_6_) *δ* 164.90 (C39 or C22), 164.82 (C39 or C22), 163.65, 161.90, 161.30, 156.44, 148.48, 137.84, 137.81, 132.14, 129.31, 129.30, 127.90, 127.87, 127.20, 125.11, 121.32, 115.35, 114.61, 112.85, 101.66 (C3), 62.39 (C32), 52.72 (CH_2_), 52.66 (CH_2_), 35.45 (C15); anal. calcd; C_23_H_21_N_3_O_4_; C, 54.71; H, 3.59; N, 17.94; found; C, 54.93; H, 3.71; N, 18.09.

#### 4.1.12 7-((1-(2-((4-Bromophenyl)amino)-2-oxoethyl)-1*H*-1,2,3-triazol-4-yl)methoxy)-*N*-((1-(2-((4-bromophenyl)amino)-2-oxoethyl)-1*H*-1,2,3-triazol-4-yl)methyl)-2-oxo-2*H*-chromene-3-carboxamide (12l)

Brown solid; yield: 72%; MP = 242–244 °C; IR (KBr, *v*_max_) 3330 (NH), 3010 (CH aromatic), 2860 (CH aliphatic), 1659 (C=O) cm^−1^; ^1^H NMR (500 MHz, DMSO-*d*_6_) *δ* 10.62 (s, 1H), 10.59 (s, 1H), 9.09 (t, *J* = 5.6 Hz, 1H), 8.88 (s, 1H), 8.32 (s, 1H), 8.04 (s, 1H), 7.94 (d, *J* = 8.8 Hz, 1H), 7.55–7.54 (m, 1H), 7.54–7.53 (m, 3H), 7.52–7.50 (m, 3H), 7.50–7.49 (m, 1H), 7.31 (d, *J* = 2.2 Hz, 1H), 7.13 (dd, *J* = 8.7, 2.3 Hz, 1H), 5.36 (s, 2H), 5.35 (s, 2H), 5.31 (s, 2H), 4.61 (d, *J* = 5.4 Hz, 2H): ^13^C NMR (126 MHz, DMSO-*d*_6_) *δ* 164.90, 164.82, 163.63, 161.88, 161.28, 156.58, 148.46, 142.01, 138.24, 138.21, 132.22, 132.20, 127.19, 121.66, 115.90, 115.87, 115.34, 114.59, 112.83, 101.65, 62.38, 52.72, 52.67, 35.29; anal. calcd; C_32_H_25_Br_2_N_9_O_6_; C, 48.57; H, 3.18; N, 15.93; found; C, 48.82; H, 3.36; N, 16.12.

#### 4.1.13 2-oxo-7-((1-(2-oxo-2-((4-(Trifluoromethyl)phenyl)amino)ethyl)-1*H*-1,2,3-triazol-4-yl)methoxy)-*N*-((1-(2-oxo-2-((4-(trifluoromethyl)phenyl)amino)ethyl)-1*H*-1,2,3-triazol-4-yl)methyl)-2*H*-chromene-3-carboxamide (12m)

Brown solid; yield: 75%; MP = 286–288 °C; IR (KBr, *v*_max_) 3325 (NH), 3053 (C–H aromatic), 2961 (CH aliphatic), 1663 (C=O) cm^−1^; ^1^H NMR (500 MHz, DMSO-*d*_6_) *δ* 11.13–10.51 (m, 2H), 9.11 (t, *J* = 5.7 Hz, 1H), 8.88 (s, 1H), 8.35 (s, 1H), 8.07 (s, 1H), 7.93 (d, *J* = 8.7 Hz, 1H), 7.78 (d, *J* = 8.3 Hz, 4H), 7.74–7.65 (m, 4H), 7.31 (d, *J* = 2.4 Hz, 1H), 7.12 (dd, *J* = 8.8, 2.4 Hz, 1H), 5.43 (s, 2H), 5.40–5.30 (m, 4H), 4.62 (d, *J* = 5.6 Hz, 2H); ^13^C NMR (126 MHz, DMSO-*d*_6_) *δ* 165.41, 165.33, 163.55, 161.82, 161.24, 156.51, 148.43, 142.36 (d, *J* = 3.2 Hz), 141.96, 132.07, 127.91, 127.22 (d, *J* = 3.3 Hz), 126.82–126.52 (m), 125.75, 125.11, 124.31 (d, *J* = 4.3 Hz), 124.05 (d, *J* = 4.4 Hz), 123.60, 119.57, 115.21, 114.53, 112.76, 101.53, 62.28, 52.69, 52.63, 35.23; anal. calcd; C_34_H_25_F_6_N_9_O_6_; C, 53.06; H, 3.27; N, 16.38; found; C, 53.21; H, 3.42; N, 16.55.

#### 4.1.14 7-((1-(2-((2,4-Dimethylphenyl)amino)-2-oxoethyl)-1*H*-1,2,3-triazol-4-yl)methoxy)-*N*-((1-(2-((2,4-dimethylphenyl)amino)-2-oxoethyl)-1*H*-1,2,3-triazol-4-yl)methyl)-2-oxo-2*H*-chromene-3-carboxamide (12n)

Brown solid; yield: 74%; MP = 232–235 °C; IR (KBr, *v*_max_) 3325 (NH), 3035 (CH aromatic), 2840 (CH aliphatic), 1666 (C=O) cm^−1^, ^1^H NMR (500 MHz, DMSO-*d*_6_) *δ* 9.74 (s, 1H), 9.69 (s, 1H), 9.10 (t, *J* = 4.9 Hz, 1H), 8.87 (s, 1H), 8.32 (s, 1H), 8.04 (s, 1H), 7.92 (d, *J* = 8.6 Hz, 1H), 7.30 (s, 1H), 7.26 (d, *J* = 7.9 Hz, 2H), 7.12 (d, *J* = 8.3 Hz, 1H), 7.04–7.00 (m, 2H), 6.99–6.93 (m, 2H), 5.38 (s, 2H), 5.36–5.31 (m, 4H), 4.61 (d, *J* = 5.1 Hz, 2H), 2.23 (s, 6H), 2.17 (s, 6H): ^13^C NMR (126 MHz, DMSO-*d*_6_) *δ* 164.42, 164.34, 163.30, 163.24, 161.49, 161.41, 160.90, 160.78, 156.22, 156.18, 148.25, 148.09, 142.04, 141.58, 134.80, 134.75, 132.99, 132.96, 131.78, 131.74, 131.63, 131.02, 114.89, 114.21, 112.41, 101.21, 80.91, 73.31, 61.97, 52.03, 51.98, 34.92, 28.75, 20.53, 17.78; anal. calcd; C_36_H_35_N_9_O_6_; C, 62.69; H, 5.12; N, 18.28; found; C, 62.90; H, 5.30; N, 18.42.

#### 4.1.15 7-((1-(2-((2,4-Dimethoxyphenyl)amino)-2-oxoethyl)-1*H*-1,2,3-triazol-4-yl)methoxy)-*N*-((1-(2-((2,4-dimethoxyphenyl)amino)-2-oxoethyl)-1*H*-1,2,3-triazol-4-yl)methyl)-2-oxo-2*H*-chromene-3-carboxamide (12o)

Brown solid; yield: 75%; MP = 239–241 °C; IR (KBr, *v*_max_) 3323 (NH), 3040 (C–H aromatic), 2960 (CH aliphatic), 1667 (C=O) cm^−1^; ^1^H NMR (500 MHz, DMSO-*d*_6_) *δ* 9.62 (s, 1H), 9.57 (s, 1H), 9.09 (t, *J* = 5.6 Hz, 1H), 8.87 (s, 1H), 8.30 (s, 1H), 8.01 (s, 1H), 7.93 (d, *J* = 8.8 Hz, 1H), 7.69 (dd, *J* = 8.8, 2.1 Hz, 2H), 7.31 (d, *J* = 2.2 Hz, 1H), 7.12 (dd, *J* = 8.7, 2.3 Hz, 1H), 6.69–6.59 (m, 2H), 6.52–6.42 (m, 2H), 5.39 (s, 2H), 5.34 (s, 4H), 4.60 (d, *J* = 5.5 Hz, 2H), 3.83–3.82 (m, 6H), 3.74–3.71 (m, 6H): ^13^C NMR (126 MHz, DMSO-*d*_6_) *δ* 164.58, 164.51, 163.65, 161.90, 161.26, 157.56, 157.54, 156.59, 151.82, 148.46, 141.98, 132.13, 127.16, 125.02, 123.85, 123.80, 120.12, 115.35, 114.60, 112.84, 104.67, 101.65, 99.42, 56.26, 55.79, 52.60, 52.55, 35.30; anal. calcd; C_36_H_35_N_9_O_10_; C, 57.37; H, 4.68; N, 16.73; found; C, 57.53; H, 4.91; N, 16.91.

#### 4.1.16 2-oxo-7-((1-(2-oxo-2-((3,4,5-Trimethoxyphenyl)amino)ethyl)-1*H*-1,2,3-triazol-4-yl)methoxy)-*N*-((1-(2-oxo-2-((3,4,5-trimethoxyphenyl)amino)ethyl)-1*H*-1,2,3-triazol-4-yl)methyl)-2*H*-chromene-3-carboxamide (12p)

Brown solid; yield: 73%; MP = 228–230 °C; IR (KBr, *v*_max_) 3330 (NH), 3020 (CH aromatic), 2960 (CH aliphatic) 1663 (C=O) cm^−1^; ^1^H NMR (500 MHz, DMSO-*d*_6_) *δ* 10.44 (s, 1H), 10.41 (s, 1H), 9.10 (t, *J* = 5.0 Hz, 1H), 8.87 (s, 1H), 8.32 (s, 1H), 8.03 (s, 1H), 7.94 (d, *J* = 8.8 Hz, 1H), 7.32 (s, 1H), 7.13 (d, *J* = 10.2 Hz, 1H), 6.94 (s, 4H), 5.35 (s, 2H), 5.33 (s, 2H), 5.28 (s, 2H), 4.61 (d, *J* = 5.0 Hz, 2H), 3.74–3.70 (m, 12H), 3.61–3.58 (m, 6H): ^13^C NMR (126 MHz, DMSO-*d*_6_) *δ* 164.15, 164.06, 163.24, 161.51, 160.92, 156.17, 152.88, 148.08, 141.64, 134.57, 134.55, 133.85, 133.82, 131.73, 126.88, 124.78, 114.86, 114.19, 112.43, 101.20, 97.06, 61.97, 60.17, 55.76, 52.34, 52.30, 34.91; anal. calcd; C_38_H_39_N_9_O_12_; C, 56.09; H, 4.83; N, 15.49; found; C, 56.26; H, 5.04; N, 15.70.

#### 4.1.17 7-((1-(2-(Benzylamino)-2-oxoethyl)-1*H*-1,2,3-triazol-4-yl)methoxy)-*N*-((1-(2-(benzylamino)-2-oxoethyl)-1*H*-1,2,3-triazol-4-yl)methyl)-2-oxo-2*H*-chromene-3-carboxamide (12q)

Cream solid; yield: 66%; MP = 215–217 °C; IR (KBr, *v*_max_) 3280 (NH), 3010 (CH aromatic), 2945 (CH aliphatic), 1664 (C=O) cm^−1^, ^1^H NMR (500 MHz, DMSO-*d*_6_) *δ* 9.08 (t, *J* = 5.5 Hz, 1H), 8.87 (s, 1H), 8.84 (d, *J* = 5.4 Hz, 1H), 8.81 (t, *J* = 5.3 Hz, 1H), 8.27 (s, 1H), 7.99 (s, 1H), 7.93 (d, *J* = 8.8 Hz, 1H), 7.35–7.30 (m, 5H), 7.29–7.23 (m, 6H), 7.12 (dd, *J* = 8.7, 2.3 Hz, 1H), 5.33 (s, 1H), 5.19 (s, 2H), 5.14 (s, 2H), 4.59 (d, *J* = 5.5 Hz, 2H), 4.31 (t, *J* = 5.4 Hz, 4H): ^13^C NMR (126 MHz, DMSO-*d*_6_) *δ* 165.56, 165.48, 163.25, 161.49, 160.91, 156.18, 148.10, 141.53, 138.79, 131.74, 128.44, 127.47, 127.15, 127.01, 126.82, 126.62, 124.63, 114.89, 114.21, 112.43, 101.21, 61.97, 51.71, 45.15, 41.63, 34.90: anal. calcd; C_34_H_31_N_9_O_6_; C, 61.72; H, 4.72; N, 19.05; found; C, 61.94; H, 4.86; N, 19.20.

#### 4.1.18 7-((1-(2-((4-Methylbenzyl)amino)-2-oxoethyl)-1*H*-1,2,3-triazol-4-yl)methoxy)-*N*-((1-(2-((4-methylbenzyl)amino)-2-oxoethyl)-1*H*-1,2,3-triazol-4-yl)methyl)-2-oxo-2*H*-chromene-3-carboxamide (12r)

Brown solid; yield: 73%; MP = 247–249 °C; IR (KBr, *v*_max_) 3328 (NH), 3055 (C–H aromatic), 2962 (CH aliphatic), 1666 (C=O) cm^−1^; ^1^H NMR (500 MHz, DMSO-*d*_6_) *δ* 9.08 (t, *J* = 5.8 Hz, 1H), 8.87 (s, 1H), 8.84–8.70 (m, 2H), 8.27 (s, 1H), 7.98 (s, 1H), 7.93 (d, *J* = 8.7 Hz, 1H), 7.30 (s, 1H), 7.24–7.02 (m, 9H), 5.33 (s, 2H), 5.18 (s, 2H), 5.12 (s, 2H), 4.60 (d, *J* = 5.6 Hz, 2H), 4.34–4.20 (m, 4H), 2.38–2.14 (m, 6H); ^13^C NMR (126 MHz, DMSO-*d*_6_) *δ* 165.37, 165.28, 163.14, 161.39, 160.81, 156.08, 148.00, 141.40, 136.10, 136.07, 135.64, 135.61, 131.63, 128.87, 127.38, 126.65, 124.54, 114.75, 114.09, 112.32, 101.09, 63.09, 61.87, 51.64, 42.15, 42.12, 34.82, 20.64; anal. calcd; C_36_H_35_N_9_O_6_; C, 62.69; H, 5.12; N, 18.28; found; C, 62.86; H, 5.30; N, 18.40.

#### 4.1.19 2-oxo-7-((1-(2-oxo-2-(Phenethylamino)ethyl)-1*H*-1,2,3-triazol-4-yl)methoxy)-*N*-((1-(2-oxo-2-(phenethylamino)ethyl)-1*H*-1,2,3-triazol-4-yl)methyl)-2*H*-chromene-3-carboxamide (12s)

Brown solid; yield: 71%; MP = 226–228 °C; IR (KBr, *v*_max_) 3339 (NH), 3032 (CH aromatic), 2866 (CH aliphatic), 1668 (C=O) cm^−1^; ^1^H NMR (400 MHz, DMSO-*d*_6_) *δ* 9.11 (t, *J* = 5.7 Hz, 2H), 8.90 (s, 1H), 8.53–8.38 (m, 4H), 8.24 (s, 1H), 8.07–7.89 (m, 2H), 7.34–7.32 (m, 1H), 7.31–7.29 (m, 1H), 7.29–7.27 (m, 1H), 7.24–7.20 (m, 6H), 5.34 (s, 2H), 5.11 (s, 2H), 5.06 (s, 2H), 4.61 (d, *J* = 5.6 Hz, 2H), 3.35–3.21 (m, 4H), 2.80–2.65 (m, 4H); ^13^C NMR (101 MHz, DMSO-*d*_6_) *δ* 165.80, 165.71, 163.63, 161.88, 157.38, 156.58, 148.67, 148.52, 141.84, 141.18, 139.64, 139.62, 132.52, 129.12, 128.85, 128.85, 127.09, 126.66, 124.91, 114.62, 112.90, 72.96, 63.53, 52.08, 40.89, 35.37; anal. calcd; C_36_H_35_N_9_O_6_; C, 62.69; H, 5.12; N, 18.28; found; C, 62.85; H, 5.32; N, 18.42.

### 4.2 Cholinesterase inhibitory activity

Butyrylcholinesterase (BChE, E.C. 3.1.1.8, from horse serum), acetylcholinesterase (AChE, E.C. 3.1.1.7, Type V-S, lyophilized powder, from electric eel, 1000 unit), acetylthiocholine iodide (ATCI), butythiocholine iodide (BTCI), 5,5-dithiobis-(2-nitrobenzoic acid) (DTNB) were provided from Sigma-Aldrich for Ellman's test. The cholinesterase-inhibitory activities of all derivatives were assessed using the modified Ellman method as previously reported. Briefly, the synthesized compounds were dissolved in DMSO and diluted in methanol, and 25 µL of the derivatives, 50 µL of the potassium phosphate buffer at pH = 8, and 25 µL of the AChE or BChE were added into each well of the 96-well plates. This plate was incubated for 15 minutes at room temperature. Afterward, 125 µL of DTNB (3 mM in buffer) and ATCI or BTCI were added, and, after 15 minutes, the absorbance at 405 nm was measured.^24,25^

### 4.3 Kinetic study

The inhibitory mode of the most potent compound, 12c, was investigated against the BChE and AChE enzymes using BTCI or ATCI (0.1–1 mM) as substrates and varying concentrations of the inhibitor.^26,27^

### 4.4 Cytotoxic assay

The cytotoxicity of the most potent analogs was assessed in SHSY-5Y cells using previously reported procedures.^28,29^

### 4.5 Molecular docking

Induced fit docking (IFD) evaluations were performed according to previously reported procedures using the Schrodinger 2018-4 suite. Briefly, the X-ray structures of AChE (PDB code: 4EY7) and BChE (PDB code: 4BDS) were prepared with the Protein Preparation Wizard interface of Maestro by removing the ligand and water molecules, adding hydrogen atoms, optimizing their position, and assigning the ionization states of acid and basic residues according to PROPKA prediction at pH 7.0. The molecular docking was performed using IFD mode.^30^ The docking grid was centered on the active site residues defined by the co-crystallized ligand, with a box size of 20 × 20 × 20 Å. Ligands were prepared using LigPrep. For each ligand, 20 poses were generated, and the top-ranking pose based on IFDScore was selected for further analysis. To validate the docking protocol, the co-crystallized ligands were redocked, and RMSD was calculated.

### 4.6 Molecular dynamics simulation

The molecular dynamics simulations for this study of 12c-AchE and 12c-BChE were performed using the Desmond v5.3 module (https://www.schrodinger.com/products/desmond) implemented in the Maestro interface (from Schrödinger 2018-4 suite) using the OPLS4 force field. The appropriate pose for the MD simulation of 12c complexes were obtained by the IFD method. To build the system for MD simulation, the protein–ligand complex was solvated with SPC explicit water molecules. The system was placed at the center of an orthorhombic box of appropriate size under periodic boundary conditions. To reflect physiological conditions, a final concentration of 0.15 M NaCl was used, and appropriate counterions were added both to neutralize the system and to simulate the ionic environment of a real cellular setting. The MD protocol involved minimization, pre-production, and MD simulation steps. In the minimization procedure, the entire system was allowed to relax for 2500 steps by the steepest descent approach. During equilibration, a positional restraint of 5 kcal mol^−1^ Å^−2^ was applied to the protein to prevent drastic structural changes while raising the temperature from 0 to 300 K. MD simulations were performed *via* NPT (constant number of atoms, constant pressure *i.e.* 1.01325 bar, and constant temperature *i.e.* 300 K) ensemble. The Nose–Hoover chain method was used as the default thermostat with a 1.0 ps interval and Martyna–Tobias–Klein as the default barostat with a 2.0 ps interval by applying an isotropic coupling style. Long-range electrostatic forces were calculated based on the particle-mesh-based Ewald approach, with the cut-off radius for coulombic forces set to 9.0 Å. Finally, the system was subjected to MD simulations using the RESPA integrator for 100 ns, with a time step of 2 fs and a box size of 10 Å. During the simulation, every 1000 ps of the actual frame was stored.

The dynamic behavior and structural changes of the systems were analyzed by the calculation of the root mean square deviation (RMSD) and RMSF. Subsequently, the energy-minimized structure calculated from the equilibrated trajectory system was evaluated to investigate each ligand–protein complex interaction.^31^

## Author contributions

S. K. and M. M. synthesized compounds and contributed to the characterization of compounds. A. I performed *in silico* study. M. F. supervised the study. All authors read and approved the final version of the article.

## Conflicts of interest

There is no conflicts to declare.

## Supplementary Material

RA-016-D5RA09311B-s001

## Data Availability

The author makes their data available upon request from Morteza Farnia. Supplementary information (SI) is available. See DOI: https://doi.org/10.1039/d5ra09311b.
