# Supplementary material for: Coumarin bearing triazole hybrids as cholinesterase inhibitors targeting Alzheimer's disease
Source: RSC Adv. 2026 Apr 20;16(22):20364–80. doi: 10.1039/d5ra09311b (PMC13093879; doi:10.1039/d5ra09311b)

Supplementary Information (SI) for RSC Advances.
This journal is © The Royal Society of Chemistry 2026

Fig. S1. ^{1}H NMR of 12a

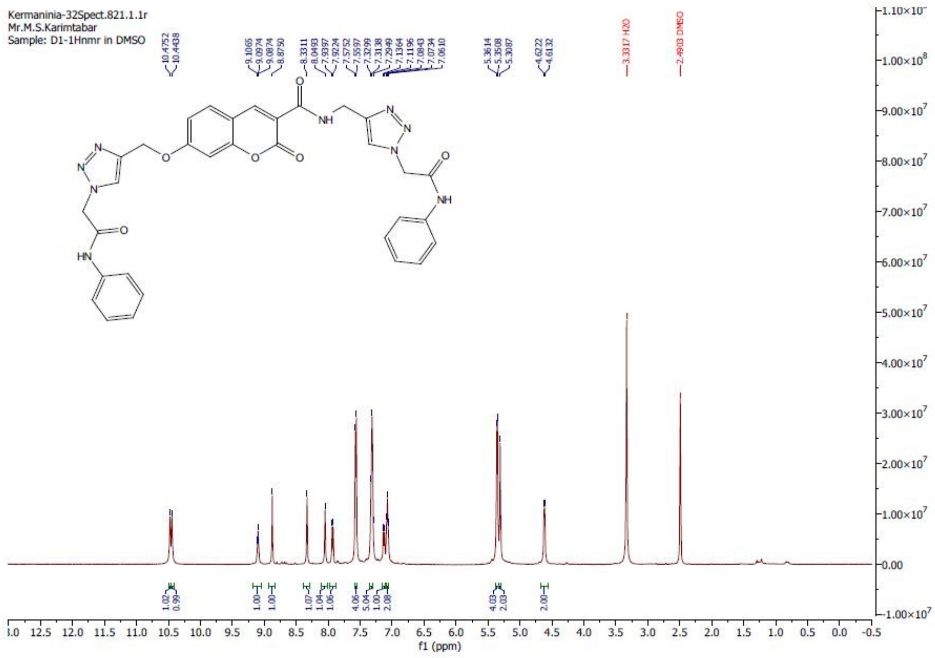

Fig. S2. ^{13}C NMR of 12a

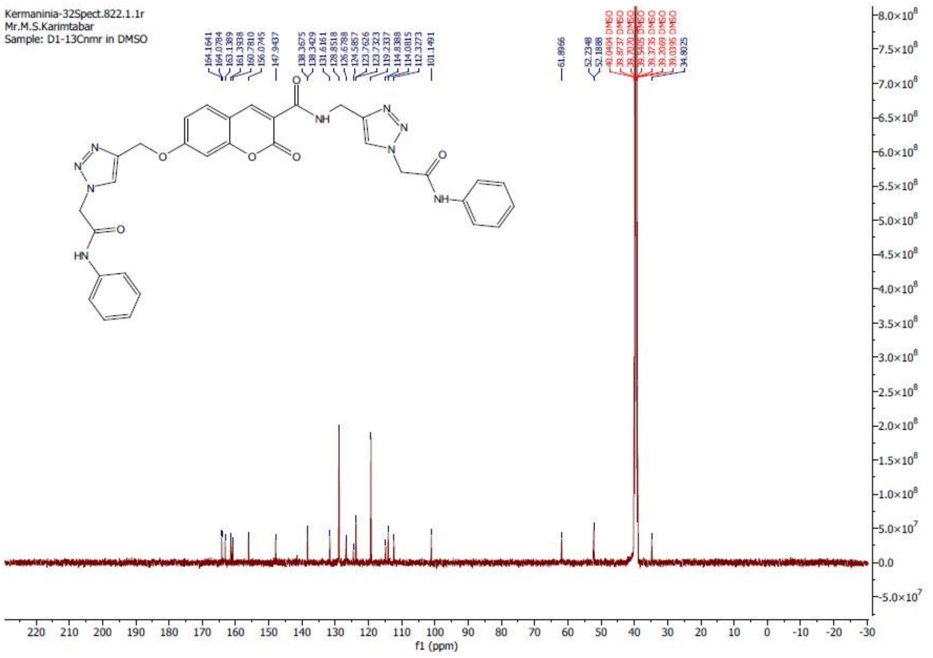

Fig. S3. ^{1}H NMR of 12b

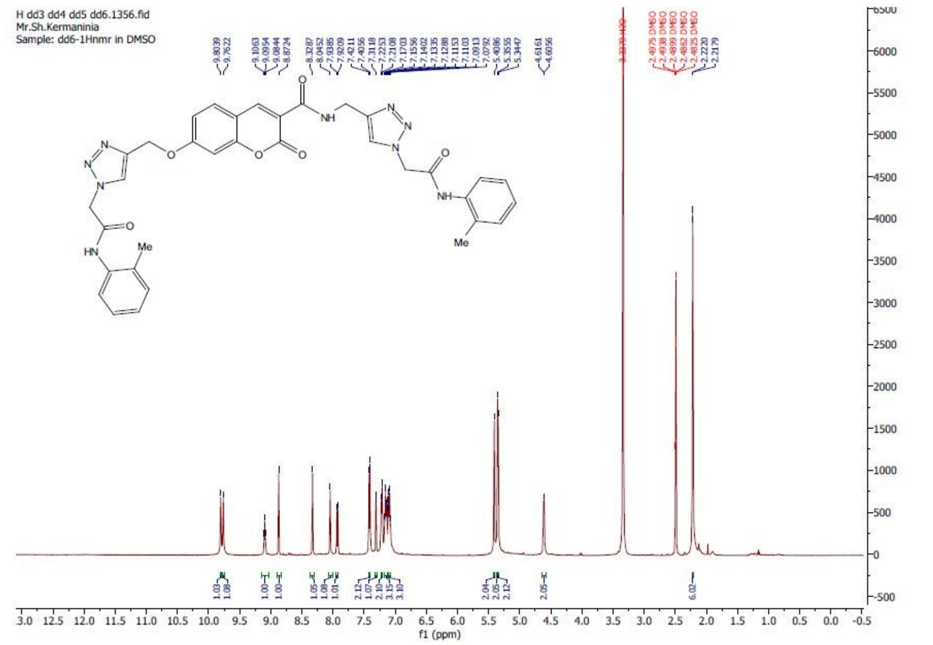

Fig. S4. ^{13}C NMR of 12b

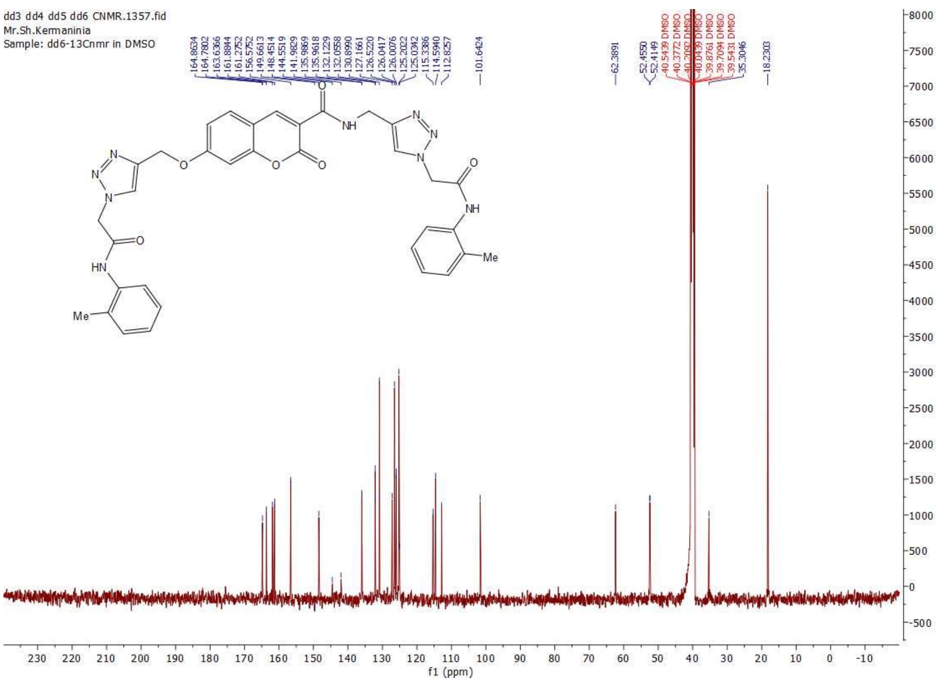

Fig. S5. ^{1}H NMR of 12c

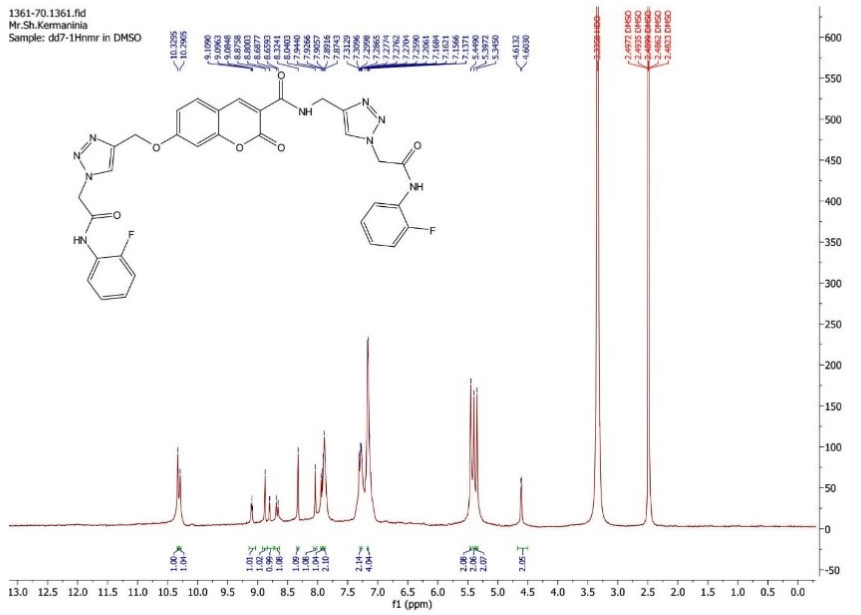

Fig. S6. ^{13}C NMR of 12c

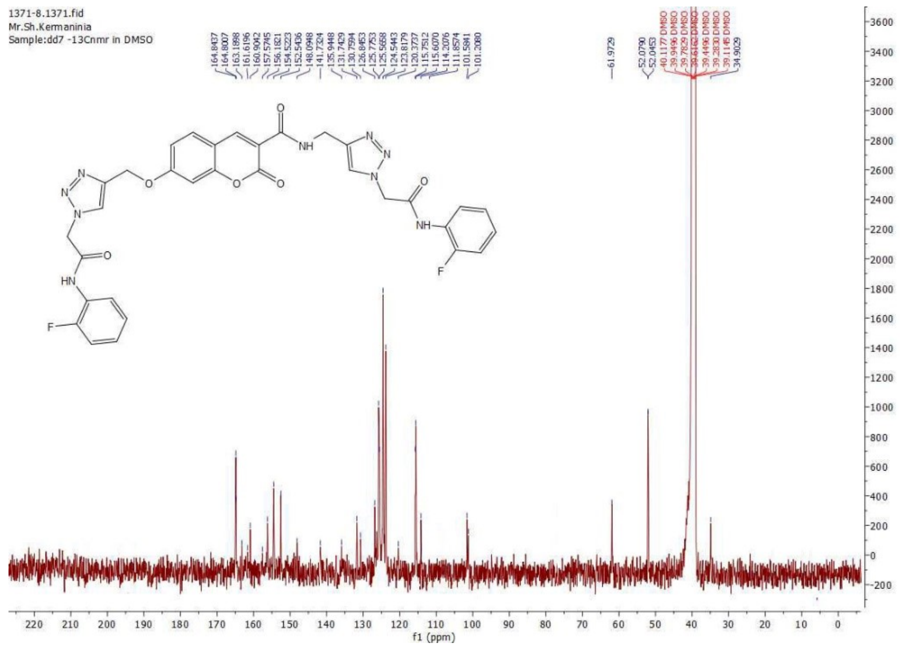

Fig. S7. HPLC of 12c

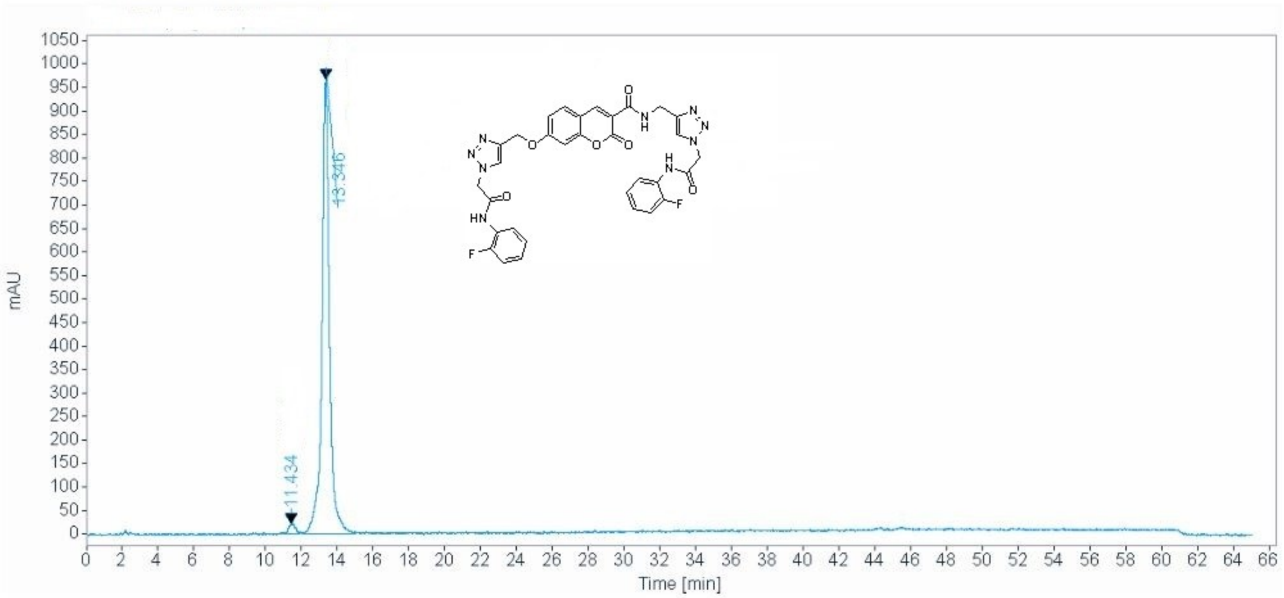

Fig. S8. ^{1}H NMR of 12d

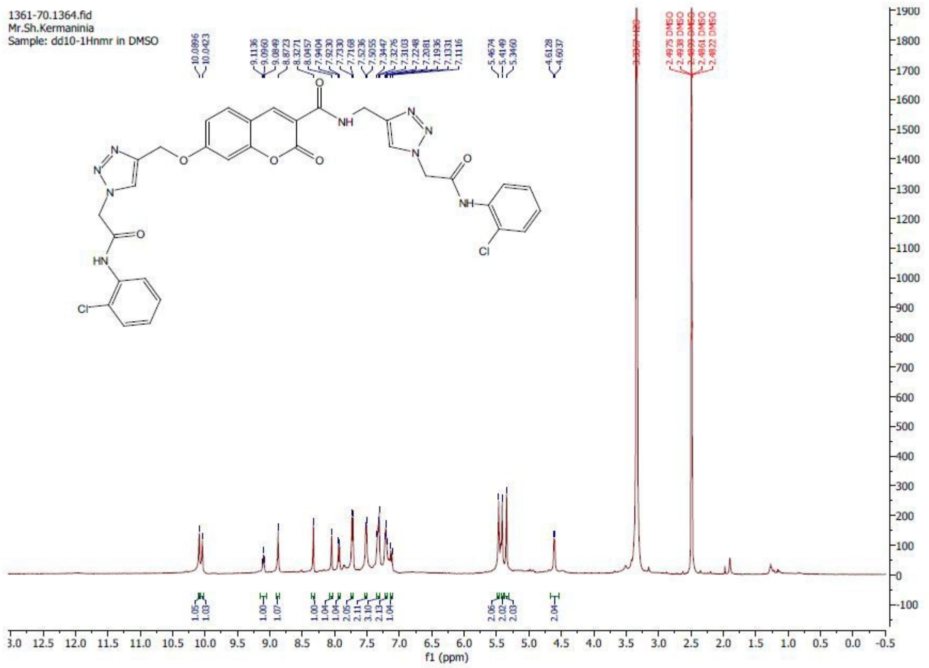

Fig. S9. ^{13}C NMR of 12d

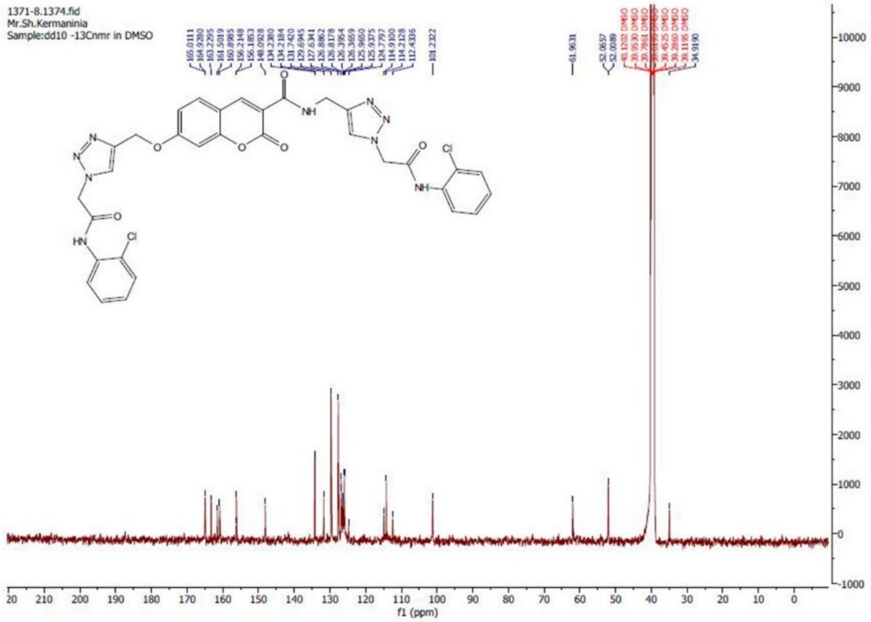

Fig. S10. ^{1}H NMR of 12e

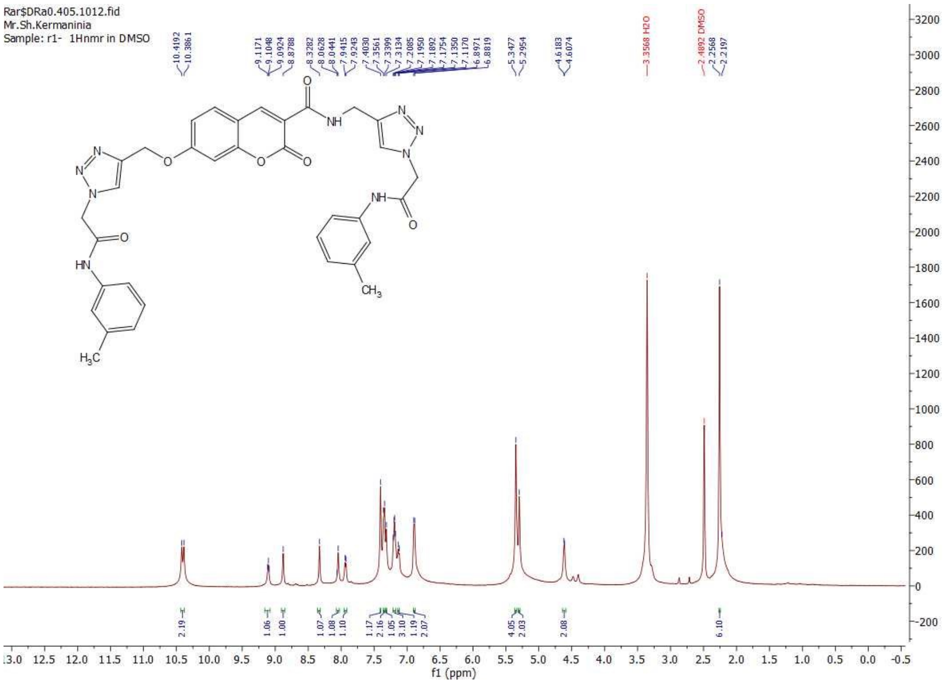

Fig. S11. ^{13}C NMR of 12e

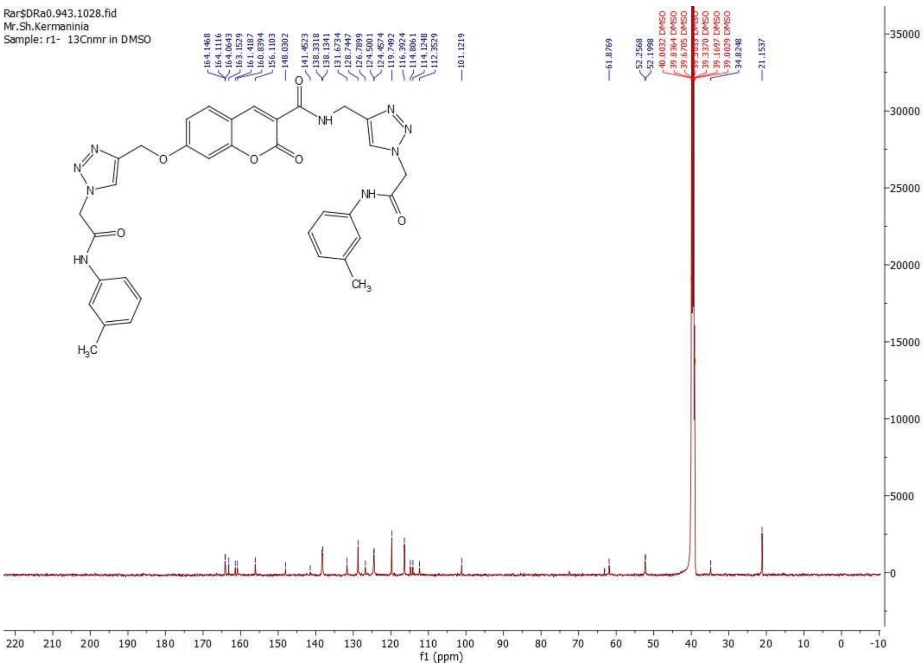

Fig. S12. ^{1}H NMR of 12f

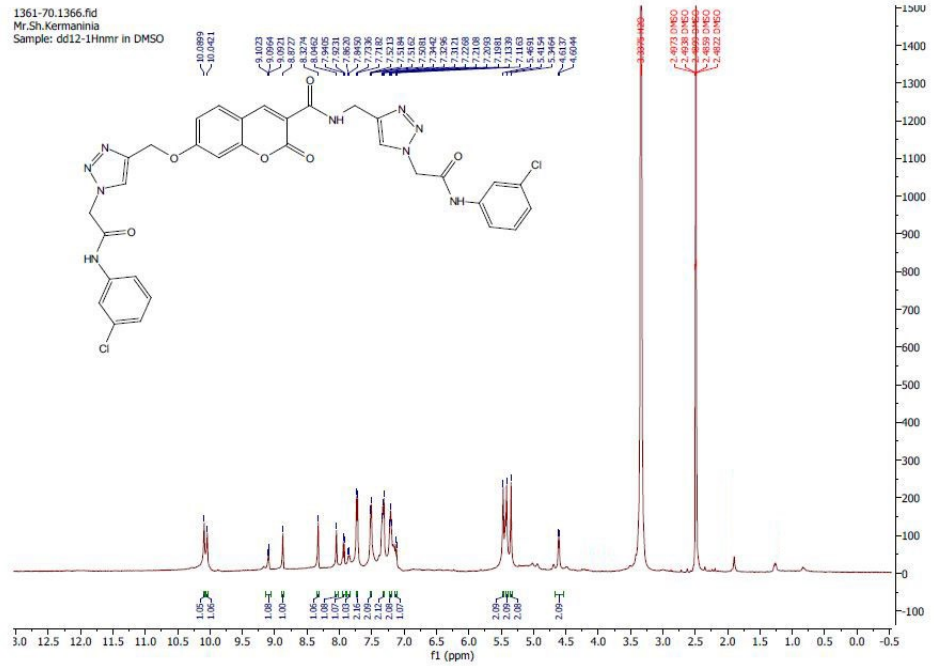

Fig. S13. ^{13}C NMR of 12f

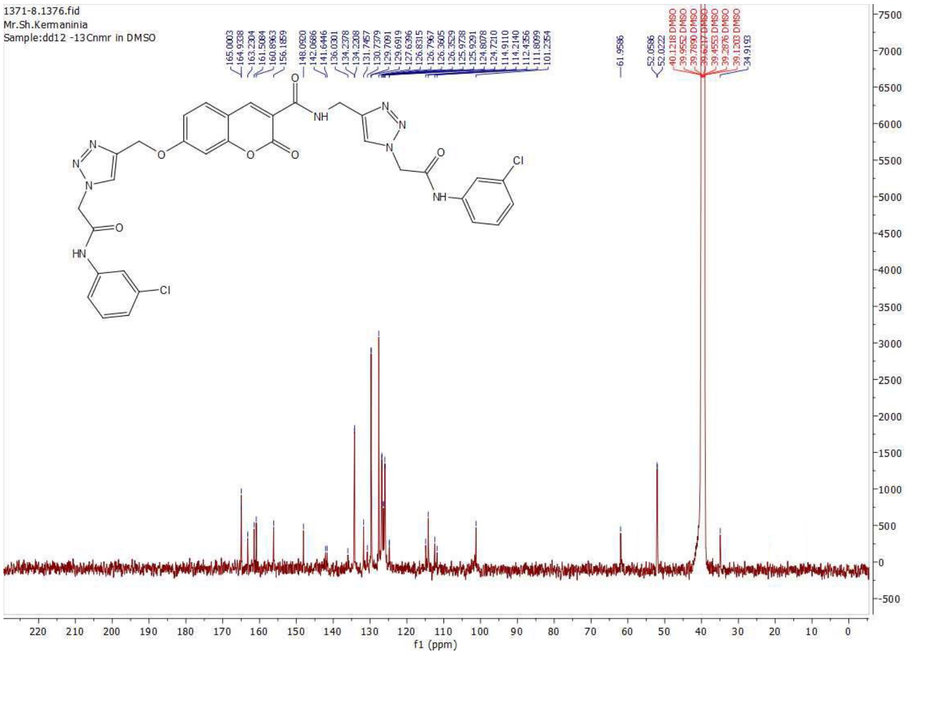

Fig. S14. ^{1}H NMR of 12g

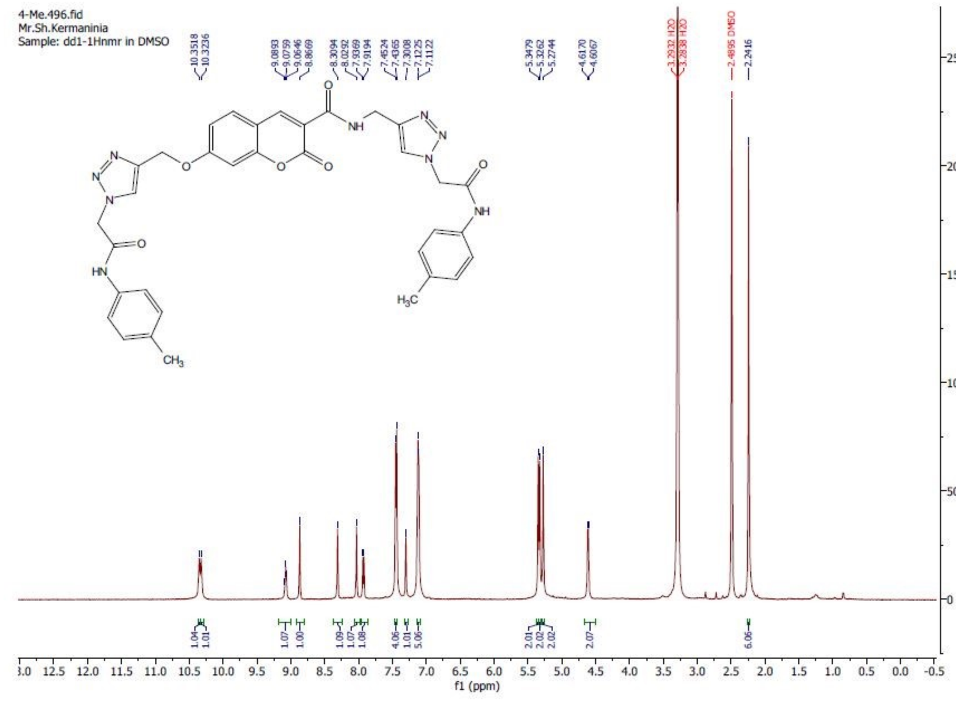

Fig. S15. ^{13}C NMR of 12g

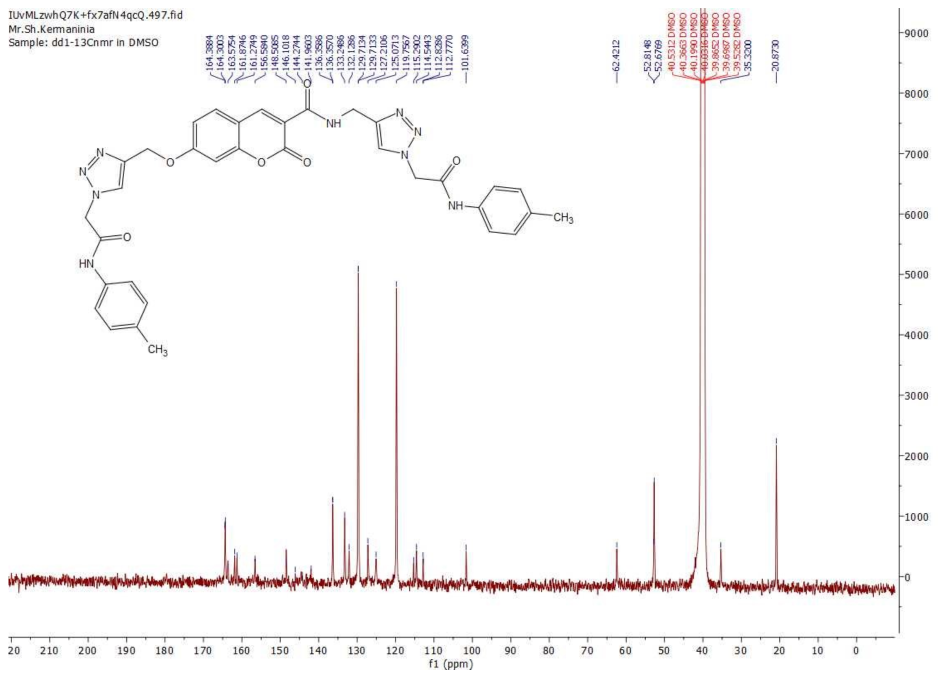

Fig. S16. HRMS of 12g

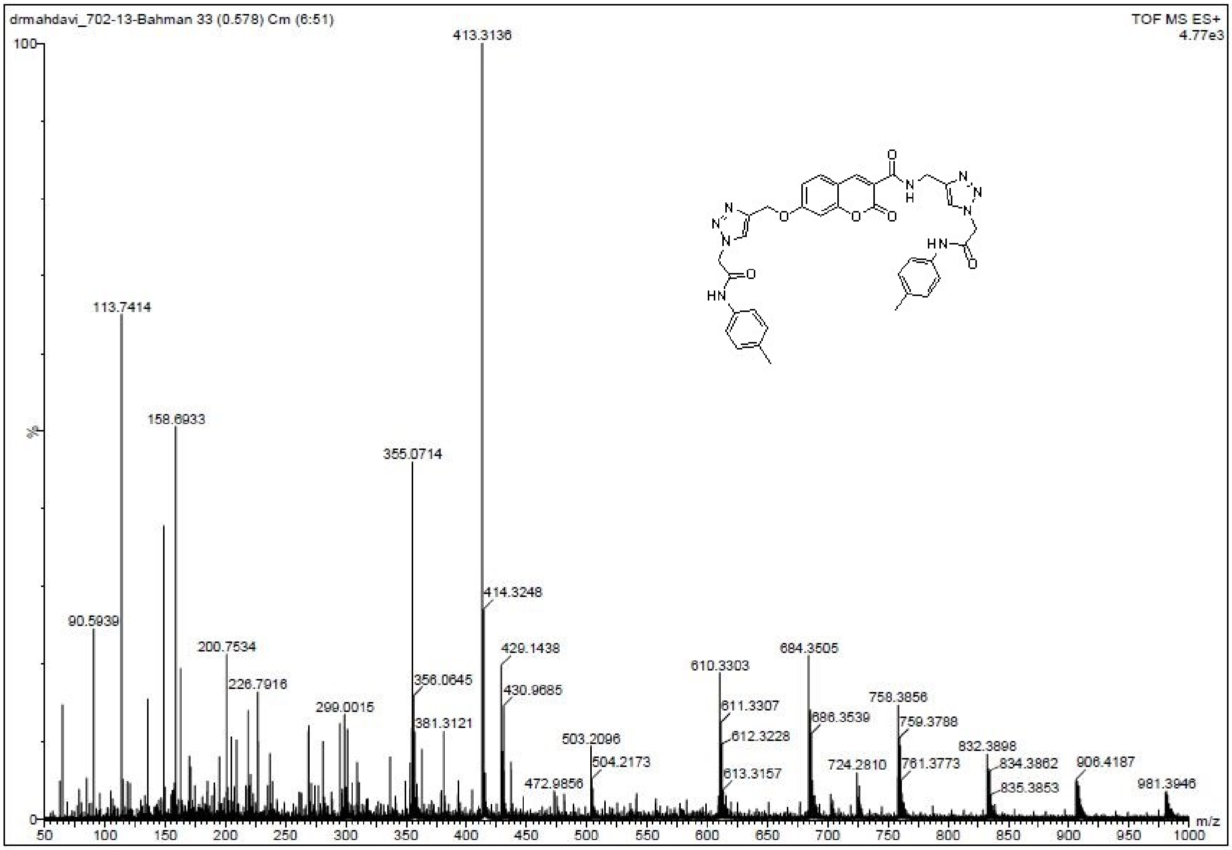

Fig. S17. ^{1}H NMR of 12h

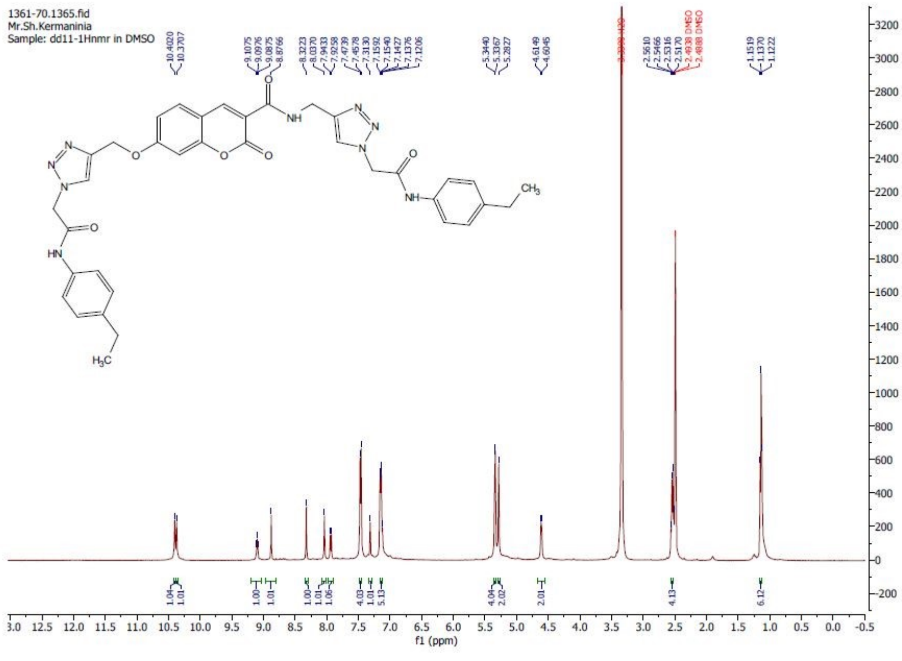

Fig. S18. ^{13}C NMR of 12h

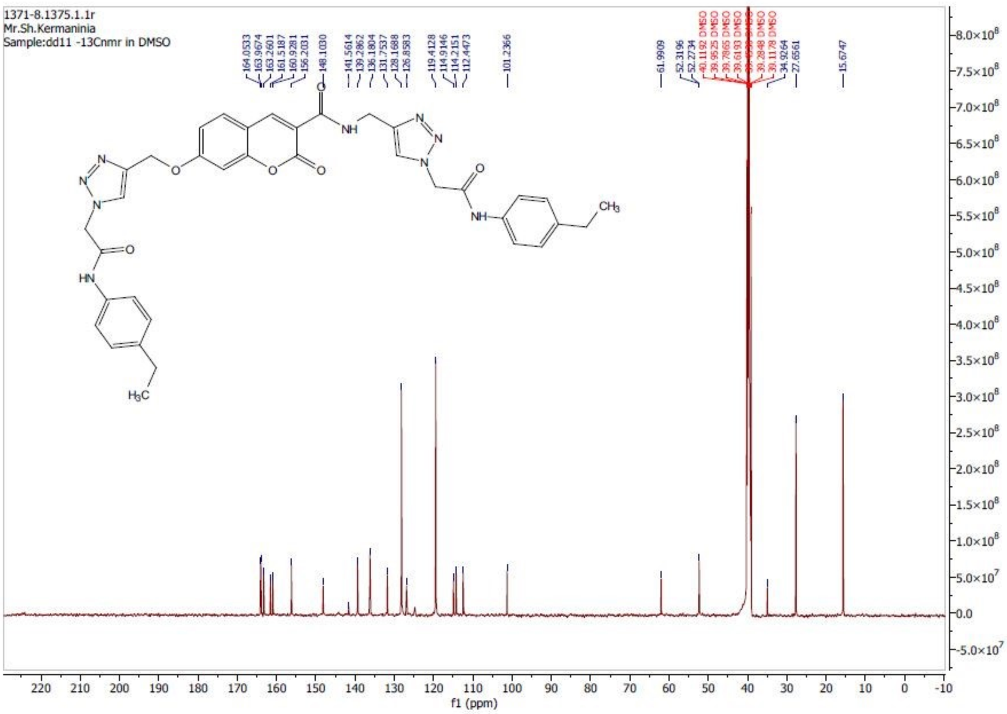

Fig. S19. ^{1}H NMR of 12i

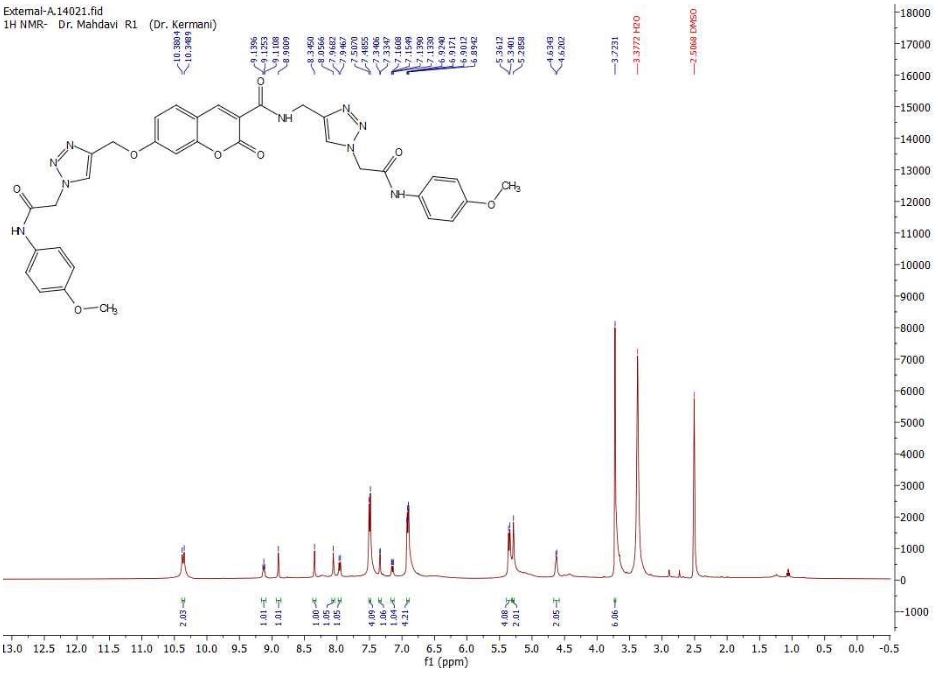

Fig. S20. ^{13}C NMR of 12i

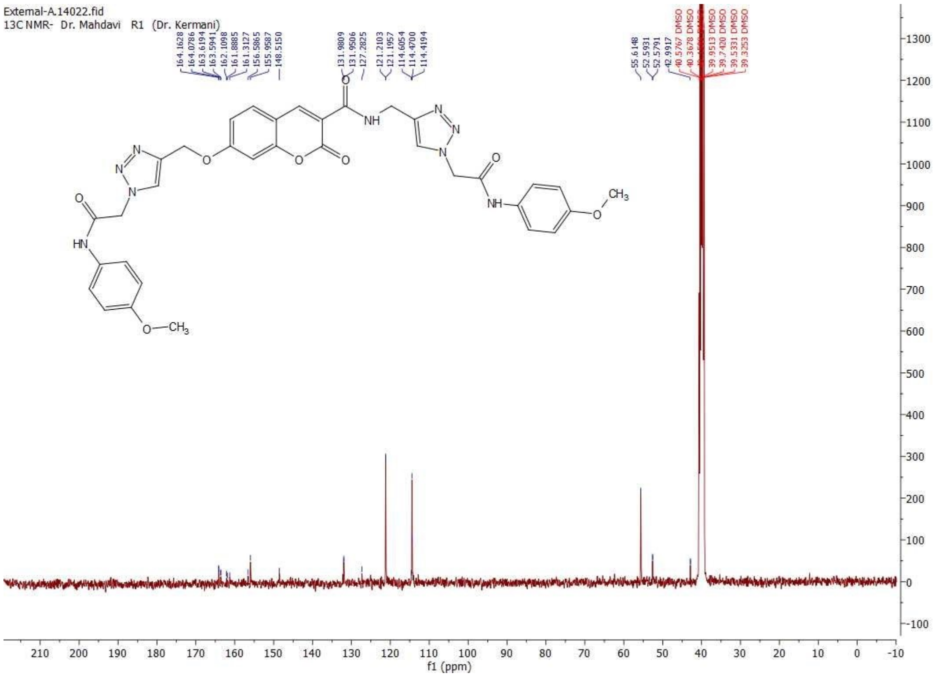

Fig. S21. ^{1}H NMR of 12j

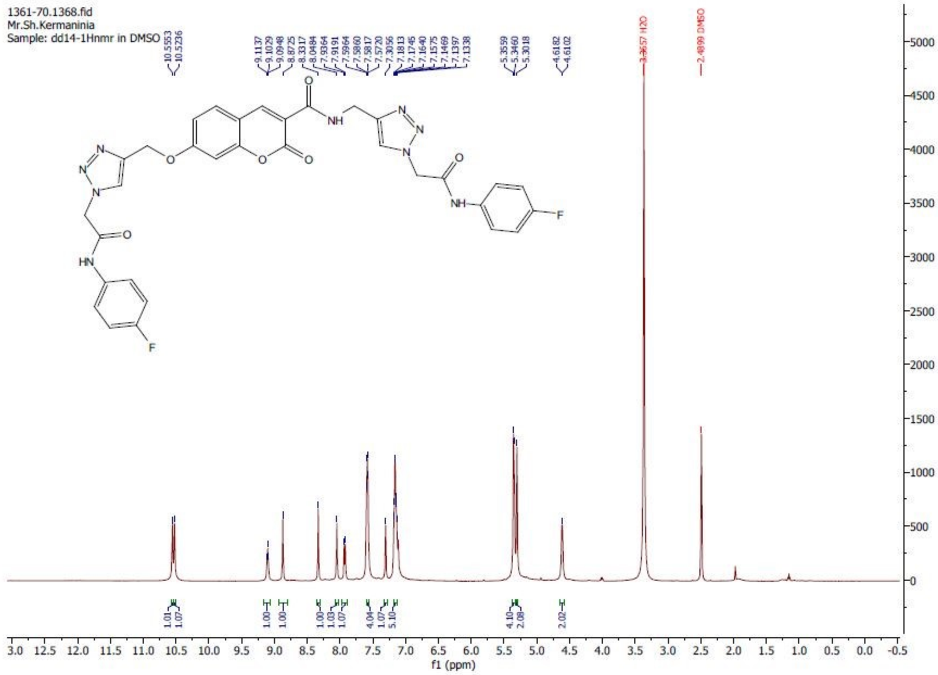

Fig. S22. ^{13}C NMR of 12j

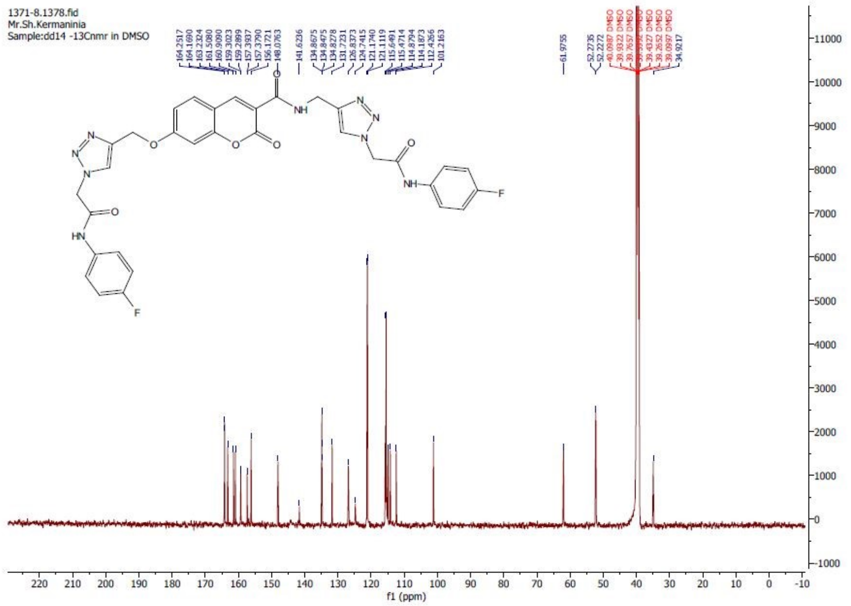

Fig. S23. ^{19}F NMR of 12j

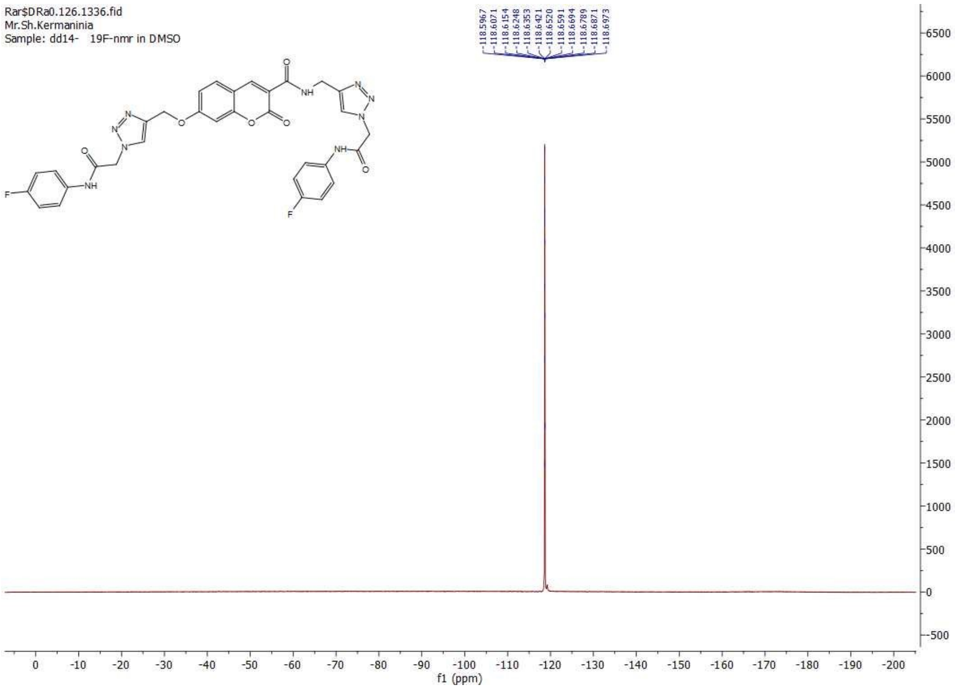

Fig. S24. ^{1}H NMR of 12k

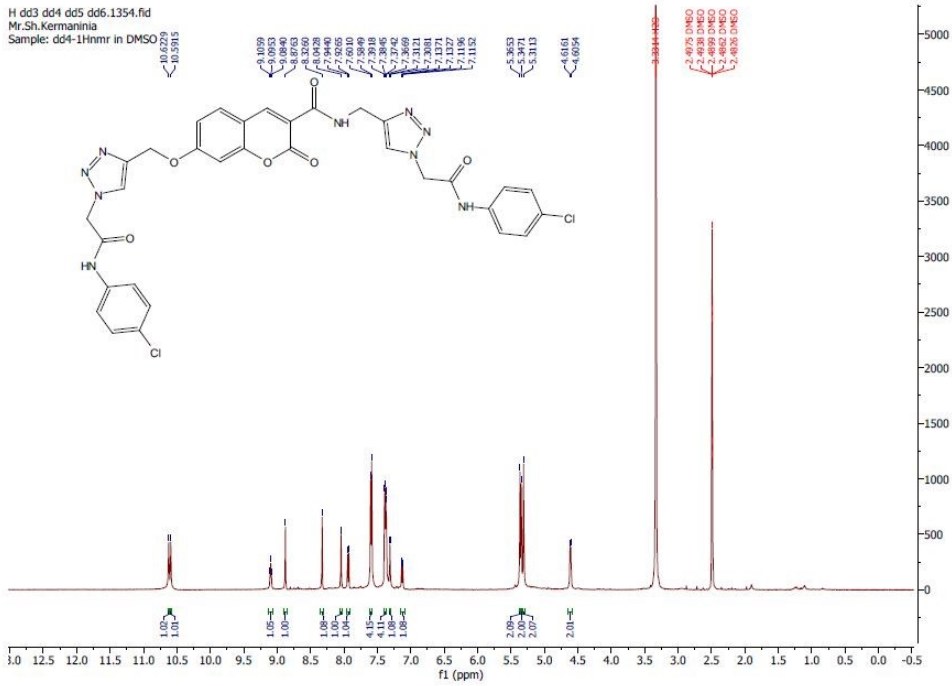

Fig. S25. ^{13}C NMR of 12k

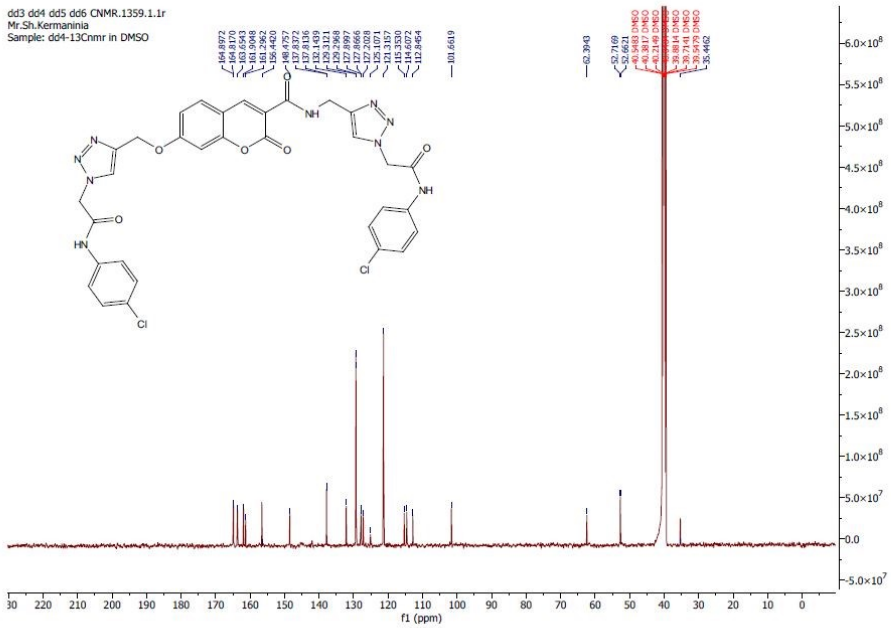

Fig. S26. ^{1}H NMR of 12l

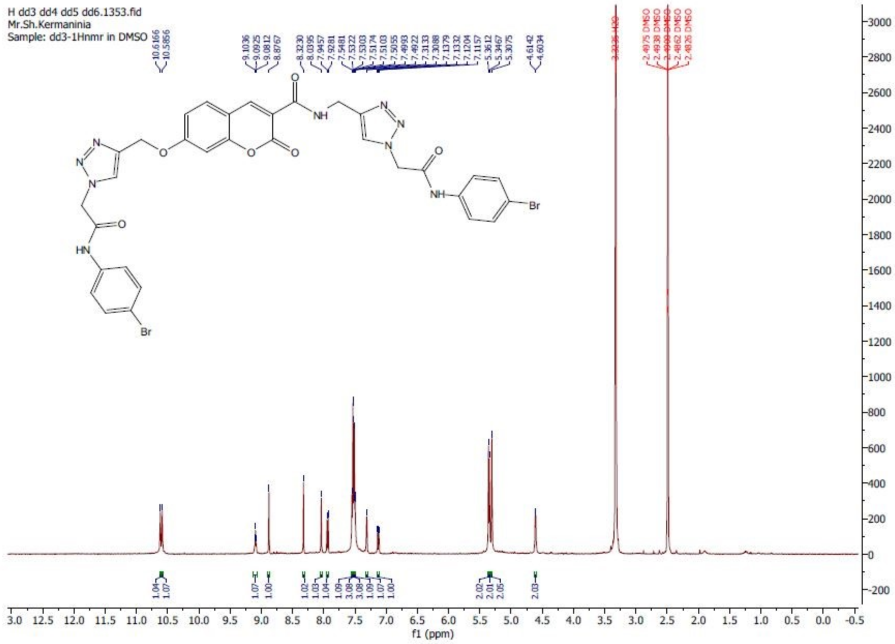

Fig. S27. ^{13}C NMR of 12l

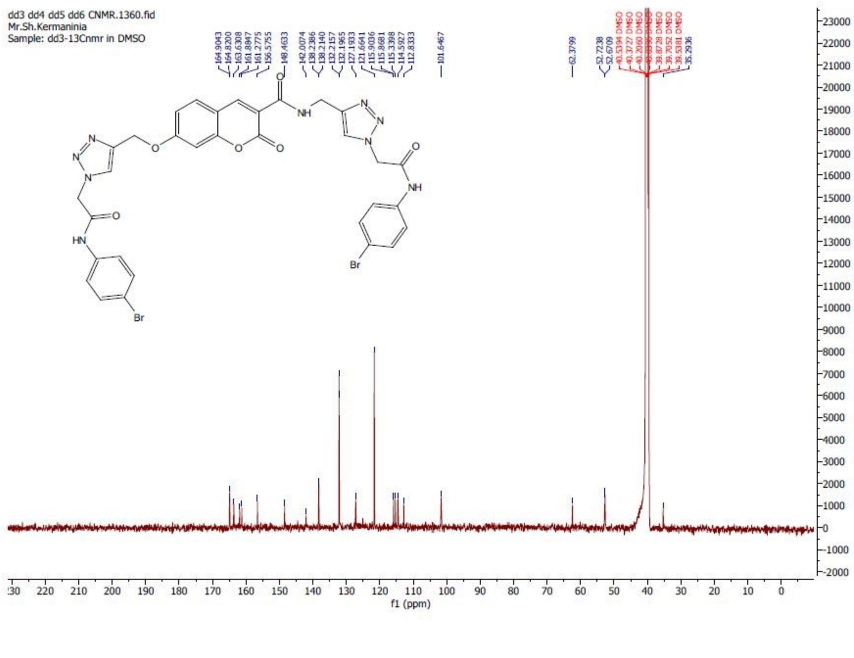

Fig. S28. ^{1}H NMR of 12m

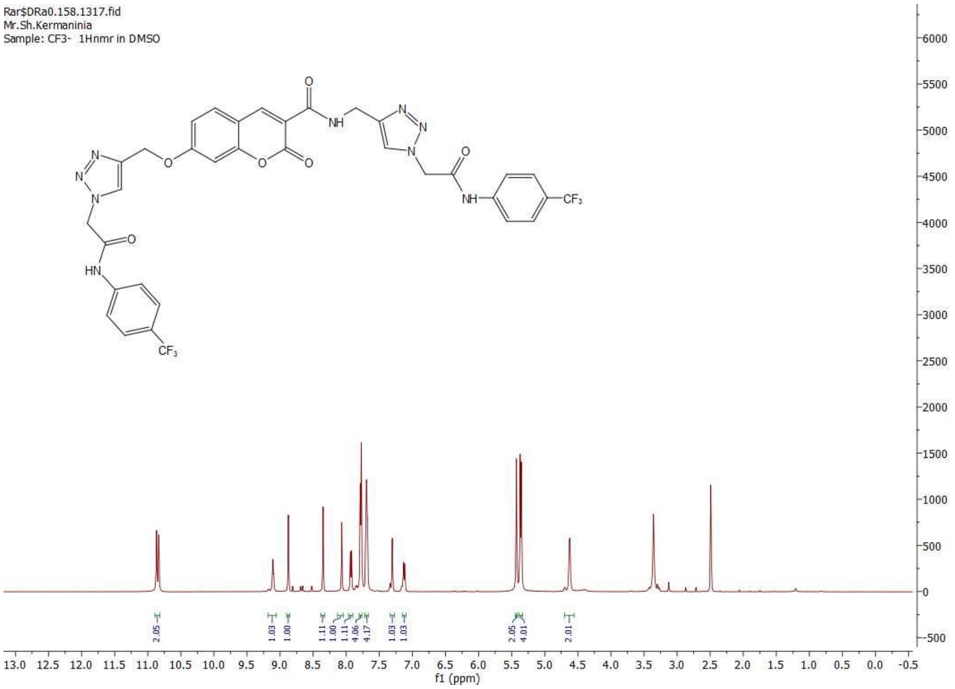

Fig. S29. ^{13}C NMR of 12m

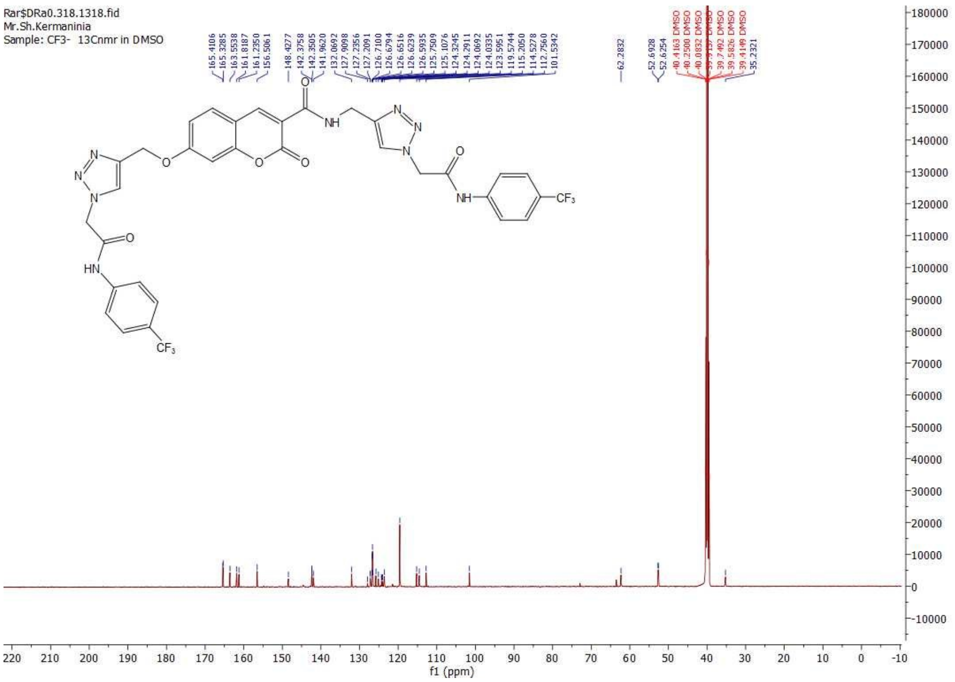

Fig. S30. ^{1}H NMR of 12n

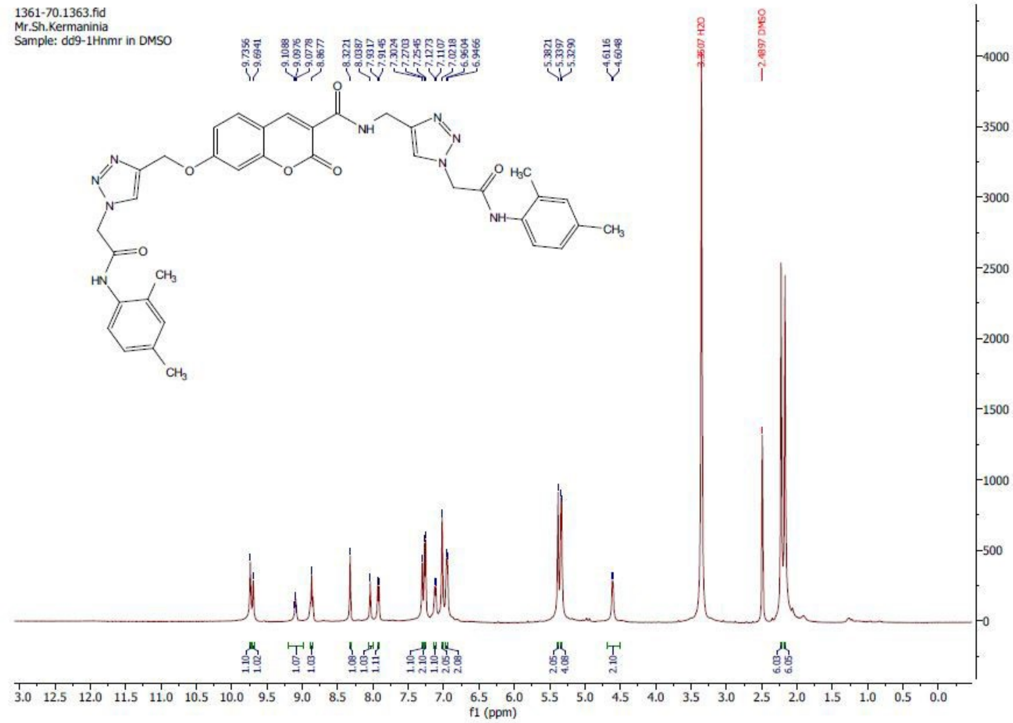

Fig. S31. ^{13}C NMR of 12n

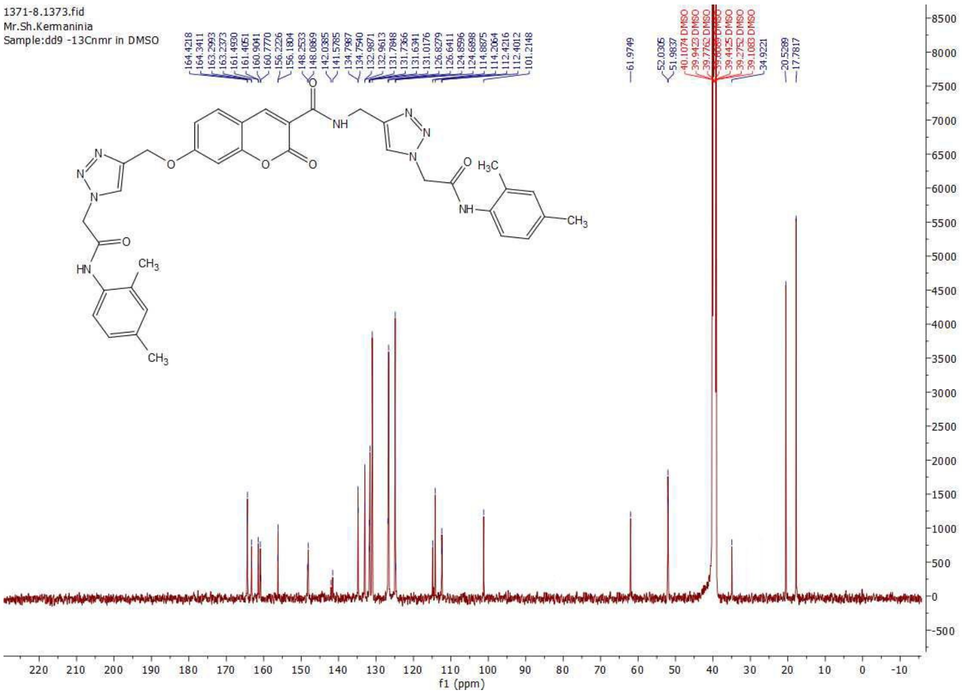

Fig. S32. ^{1}H NMR of 12o

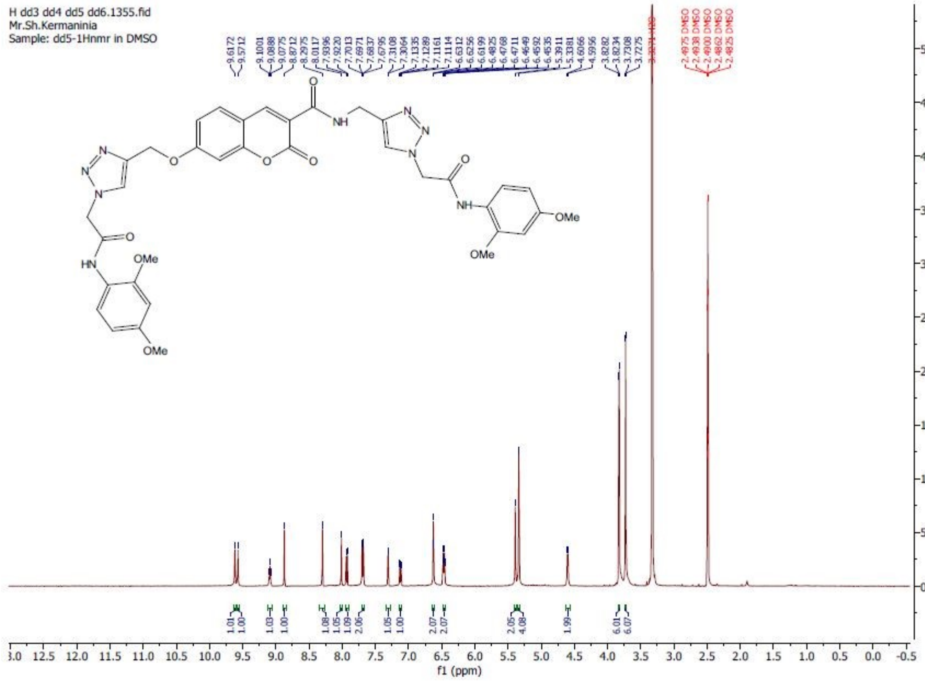

Fig. S33. ^{13}C NMR of 12o

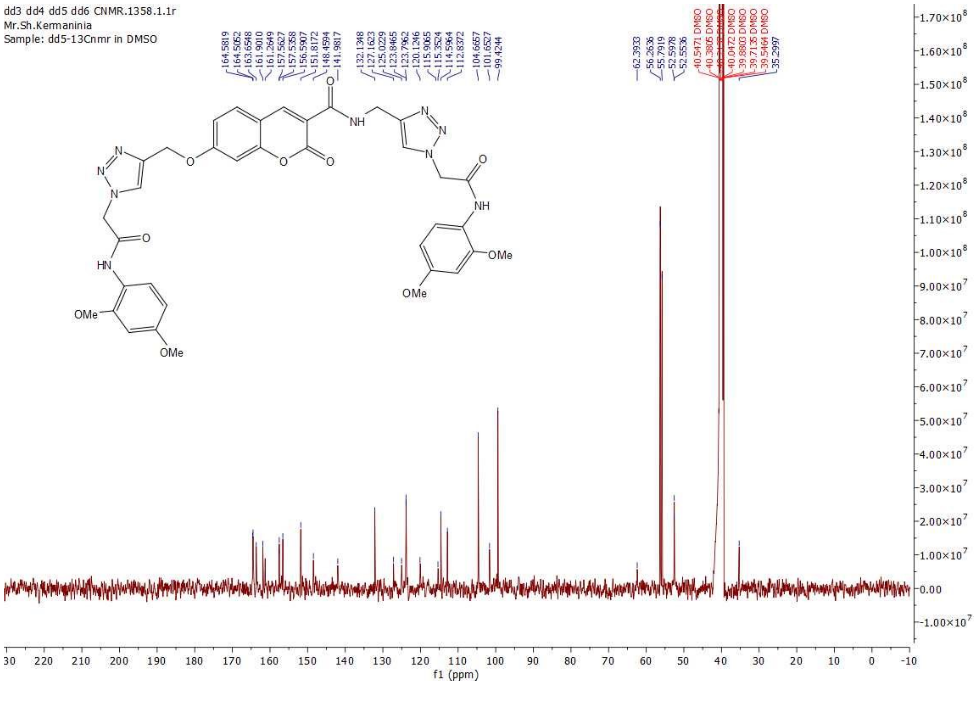

Fig. S34. ^{1}H NMR of 12p

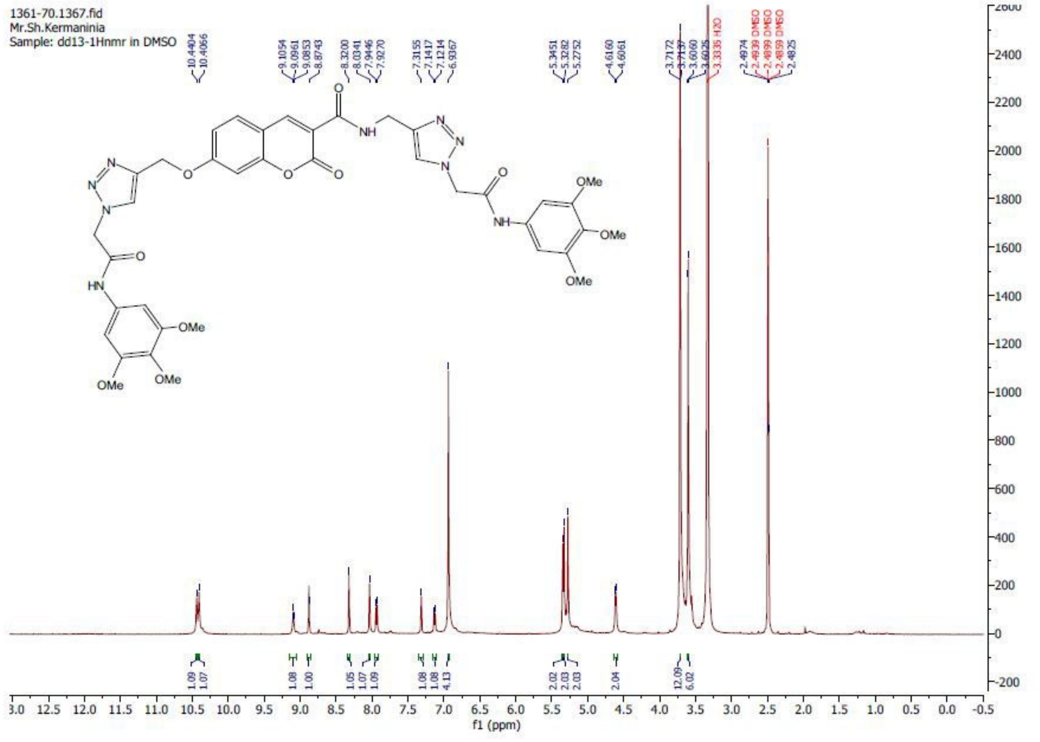

Fig. S35. ^{13}C NMR of 12p

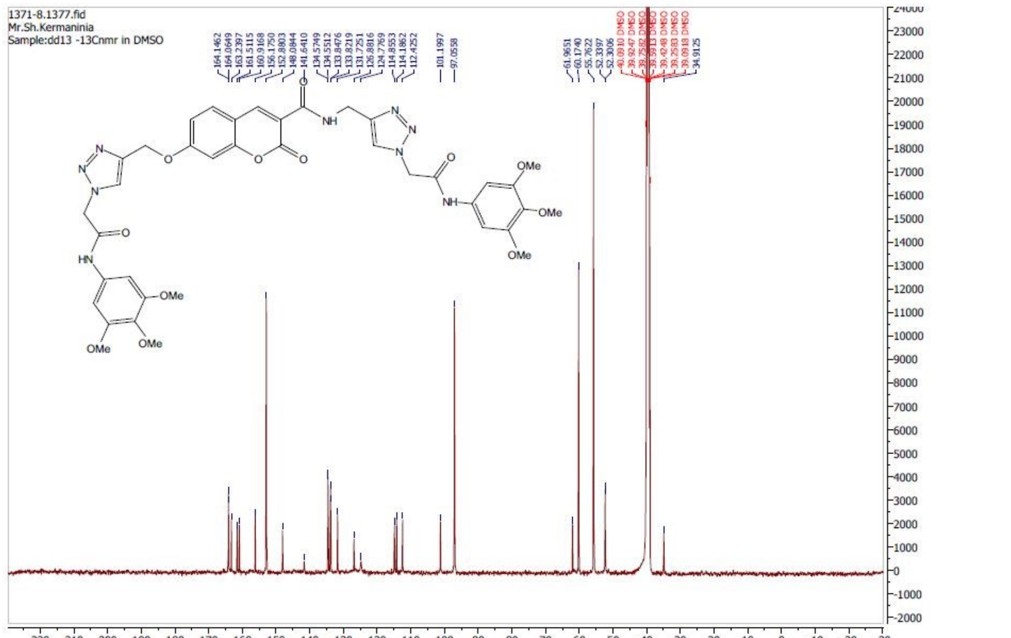

Fig. S36. ^{1}H NMR of 12q

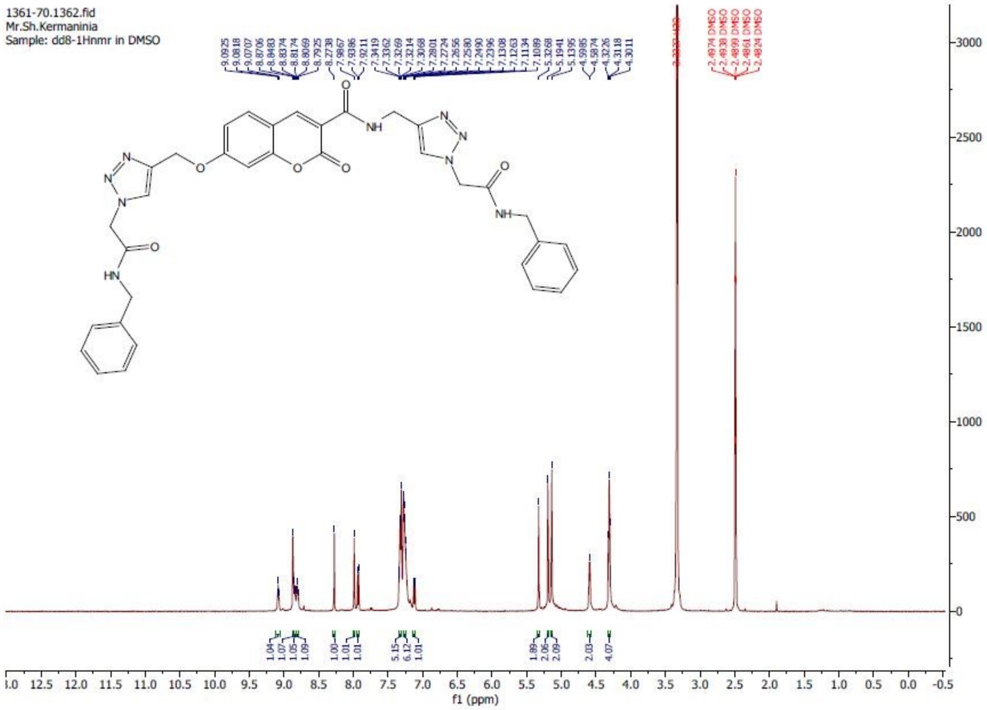

Fig. S37. ^{13}C NMR of 12q

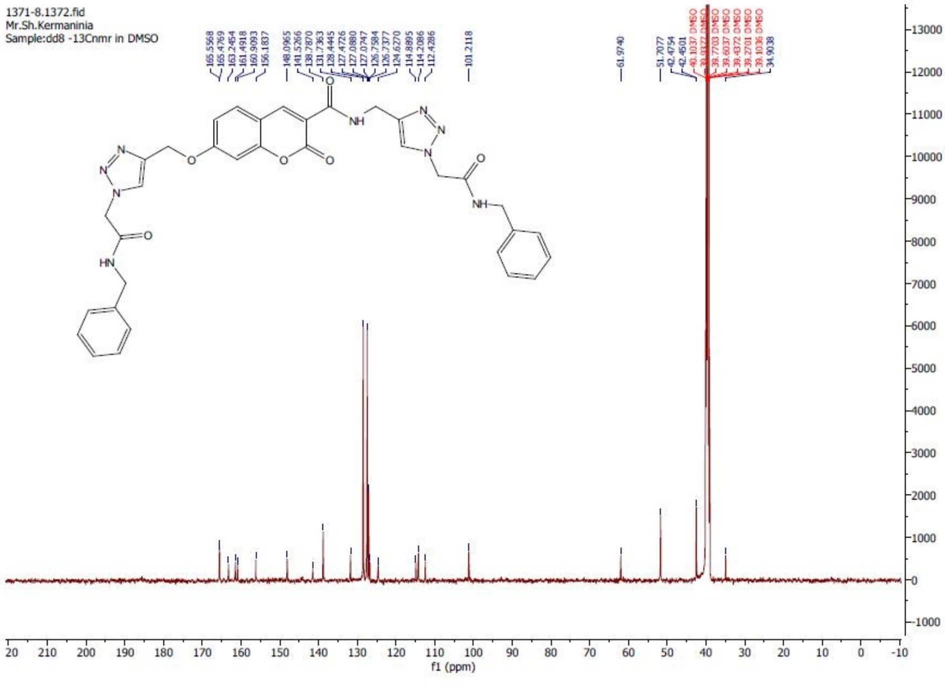

Fig. S38. ^{1}H NMR of 12r

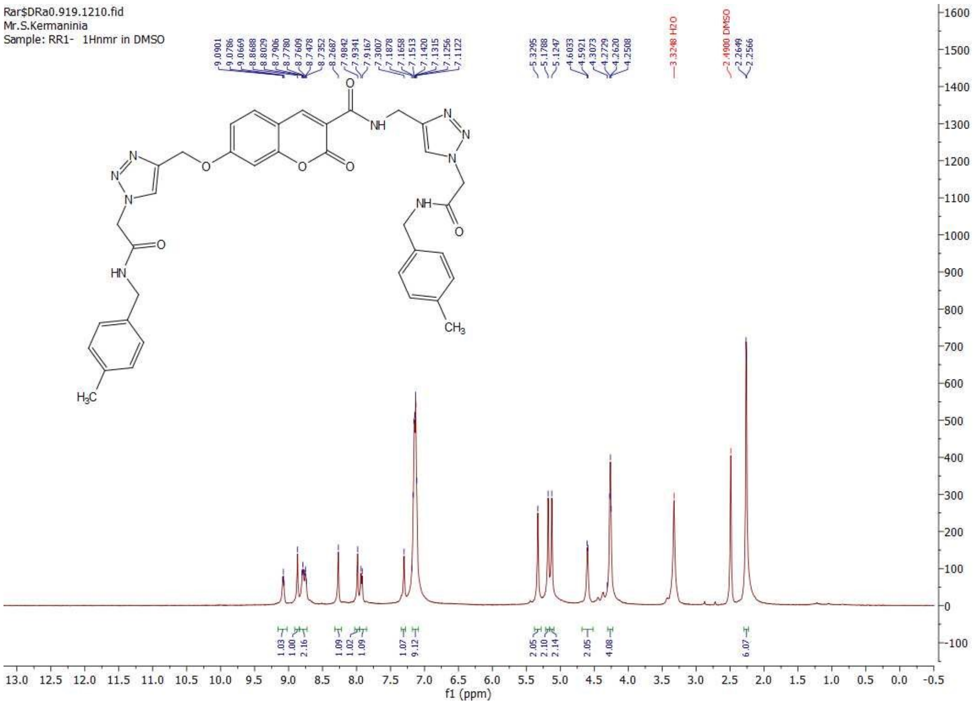

Fig. S39. ^{13}C NMR of 12r

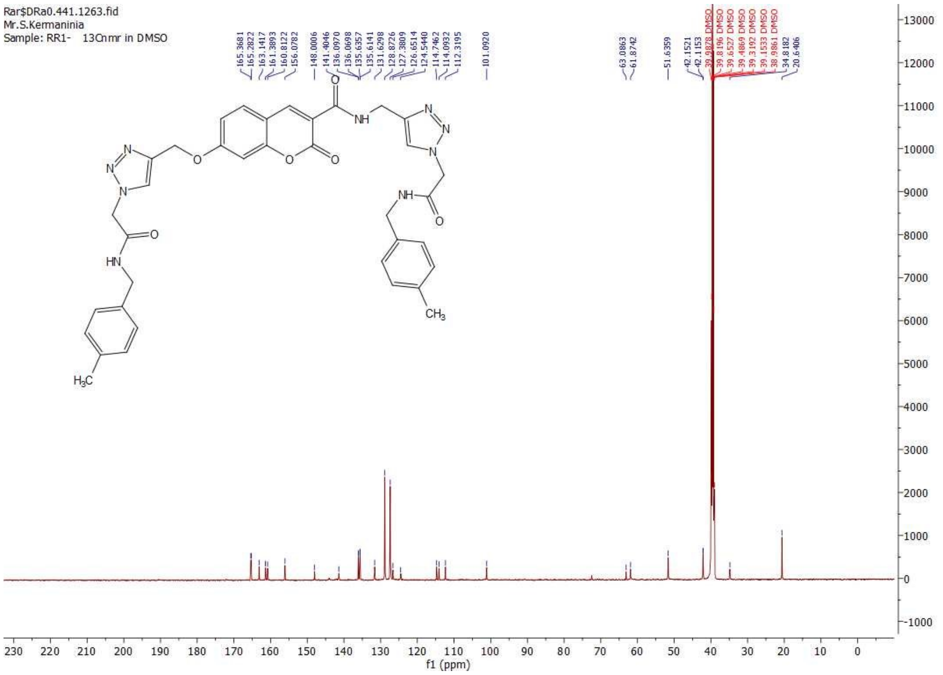

Fig. S40. ^{1}H NMR of 12s

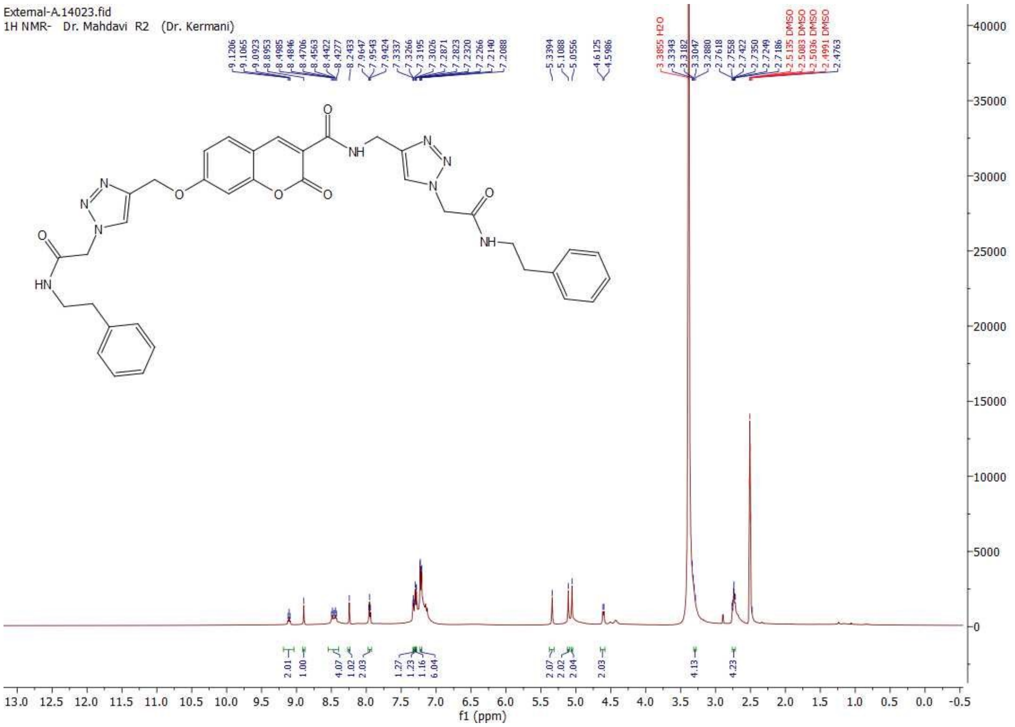

Fig. S41. ^{13}C NMR of 12s

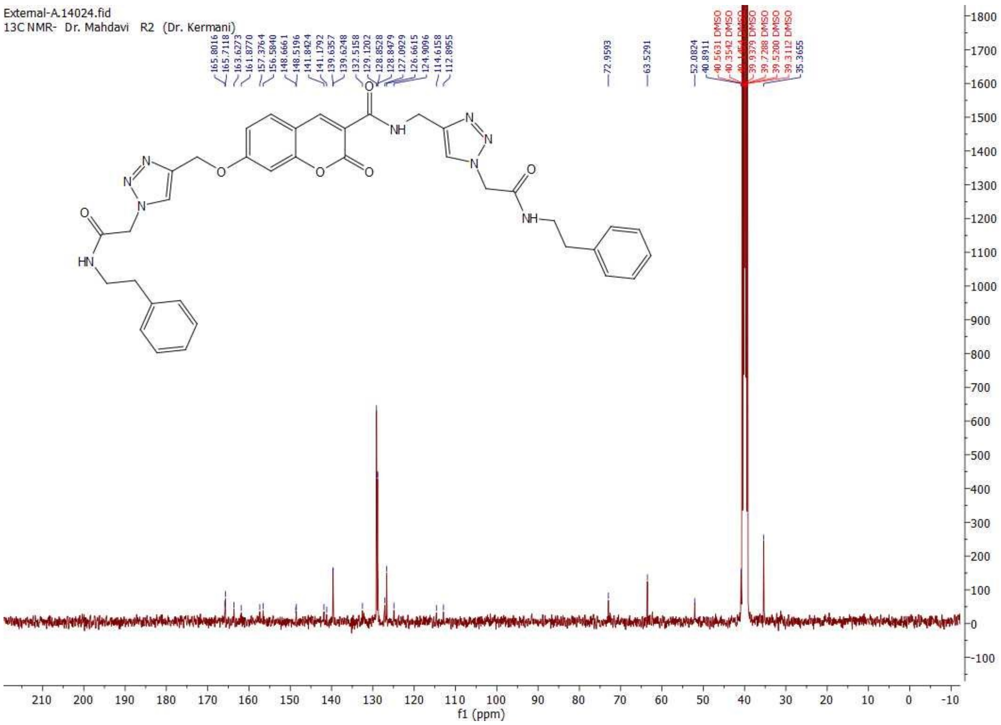

Supplement: RA-016-D5RA09311B-s001 [file RA-016-D5RA09311B-s001.pdf]
